# Hepatoprotective Evidence for the Individual Ingredients of a Standardized Sri Lankan Polyherbal Formulation: A Scoping Review

**DOI:** 10.7759/cureus.100029

**Published:** 2025-12-24

**Authors:** Geethma J Jayawardhana, Geeshani Somaratne, Kalmee P Kariyawasam, Ananda Chandrasekara

**Affiliations:** 1 Department of Food Science and Technology, Faculty of Agriculture, University of Peradeniya, Peradeniya, LKA; 2 Department of Nutrition and Dietetics, Wayamba University of Sri Lanka, Makadura, LKA

**Keywords:** fatty liver disease, hepatoprotection, herbal pharmacology, inflammation, insulin resistance, liver enzymes, oxidative stress, polyherbal formulation

## Abstract

Chronic liver disease is a major global health burden and is increasingly driven by metabolic dysfunction, oxidative stress, inflammation, and fibrosis. Conventional therapy and lifestyle modification often achieve suboptimal adherence and incomplete reversal of hepatic injury, which has intensified interest in adjunctive hepatoprotective agents of botanical origin. A standardized Sri Lankan polyherbal formulation (LivosBEE™), combining *Osbeckia octandra, Tamarindus indica, Piper nigrum, Piper longum, Cinnamomum verum,* and multifloral bee honey, is traditionally used in the management of jaundice, fatty liver, and hepatocellular inflammation. However, its evidence base has not been comprehensively mapped.

A scoping review was conducted in accordance with the Preferred Reporting Items for Systematic reviews and Meta-Analyses extension for Scoping Reviews (PRISMA-ScR) and JBI methodological guidance to identify preclinical and clinical studies reporting liver-protective effects of the individual ingredients (*O. octandra*, *T. indica*, *P. nigrum*, *P. longum*, *C. verum*, and bee honey). Major electronic databases (including PubMed, Scopus, and Web of Science) and relevant grey literature were searched without date restriction. Eligible studies included in vitro experiments, in vivo animal models, and human trials that evaluated any single constituent of the formulation with respect to hepatocellular outcomes (e.g., serum aminotransferases, oxidative stress markers, lipid metabolism, histopathology, steatosis, fibrosis, and inflammatory signaling) or clinical/biochemical indicators of liver function. Data were charted for study design, model, intervention, dose, comparators, mechanistic endpoints, and safety findings. A total of 85 studies met the inclusion criteria.

Evidence indicates that the ingredients possess hepatoprotective properties, including modulation of liver enzymes, reduction of oxidative stress markers, inhibition of inflammatory pathways, and protection against chemically induced liver injury. Limited clinical data were available for some ingredients, and the synergistic potential of combining these ingredients is yet to be investigated. On the whole, however, this scoping review demonstrates that the ingredients of this polyherbal formulation have scientific support for hepatoprotective effects, providing a preliminary evidence base for its development. Future research should focus on human studies, optimal dosing, and mechanistic investigations to establish efficacy and safety.

## Introduction and background

Liver diseases represent a significant global health burden, resulting in increased morbidity and mortality. Chronic hepatitis, non-alcoholic fatty liver disease (NAFLD), and drug-related liver injury are primarily driven by oxidative stress, inflammation, and hepatocellular injury, leading to gradual liver dysfunction [1,2]. Worldwide, NAFLD has emerged as the most prevalent chronic liver disease, which affects approximately 15-30% of the general population. Among individuals with obesity or type 2 diabetes, prevalence rates are significantly higher, ranging 70-80%, highlighting its strong association with metabolic disorders [3]. According to the WHO, chronic liver diseases account for approximately two million deaths annually, underscoring the urgent need for effective preventive and therapeutic strategies. Although national-level data in Sri Lanka remains limited, regional studies indicate that NAFLD is a major contributor to abnormal liver enzyme levels in both urban and rural populations [4]. In this review, the term NAFLD is used when directly referring to published studies that have adopted this terminology. However, for consistency with the current global consensus, the updated term metabolic dysfunction-associated fatty liver disease (MAFLD) is acknowledged and used throughout the text where appropriate.

Traditional therapeutic approaches, including hepatoprotective medications and lifestyle modifications, often yield suboptimal outcomes and may be accompanied by adverse effects or poor patient compliance [5]. These limitations highlight the need for more effective and safer alternatives. These challenges have stimulated growing scientific interest in complementary and plant-based hepatoprotective strategies

Polyherbal formulations combine bioactive components of multiple plants that may act synergistically to protect liver tissue through antioxidant, anti-inflammatory, and anti-apoptotic pathways. These formulations are expected to exert hepatoprotective effects through synergistic effects that promote antioxidant action, decrease inflammatory pathways, and inhibit apoptosis of hepatocytes. Several preclinical and limited clinical studies have demonstrated that individual herbal components, particularly those rich in flavonoids, polyphenols, and alkaloids, can protect liver tissue from oxidative and toxic insults, restore antioxidant enzyme balance, and improve hepatic biochemical parameters [1,6,7]. Yet, the translation of these effects from single-ingredient studies to complex polyherbal formulations remains limited.

**Figure 1:**
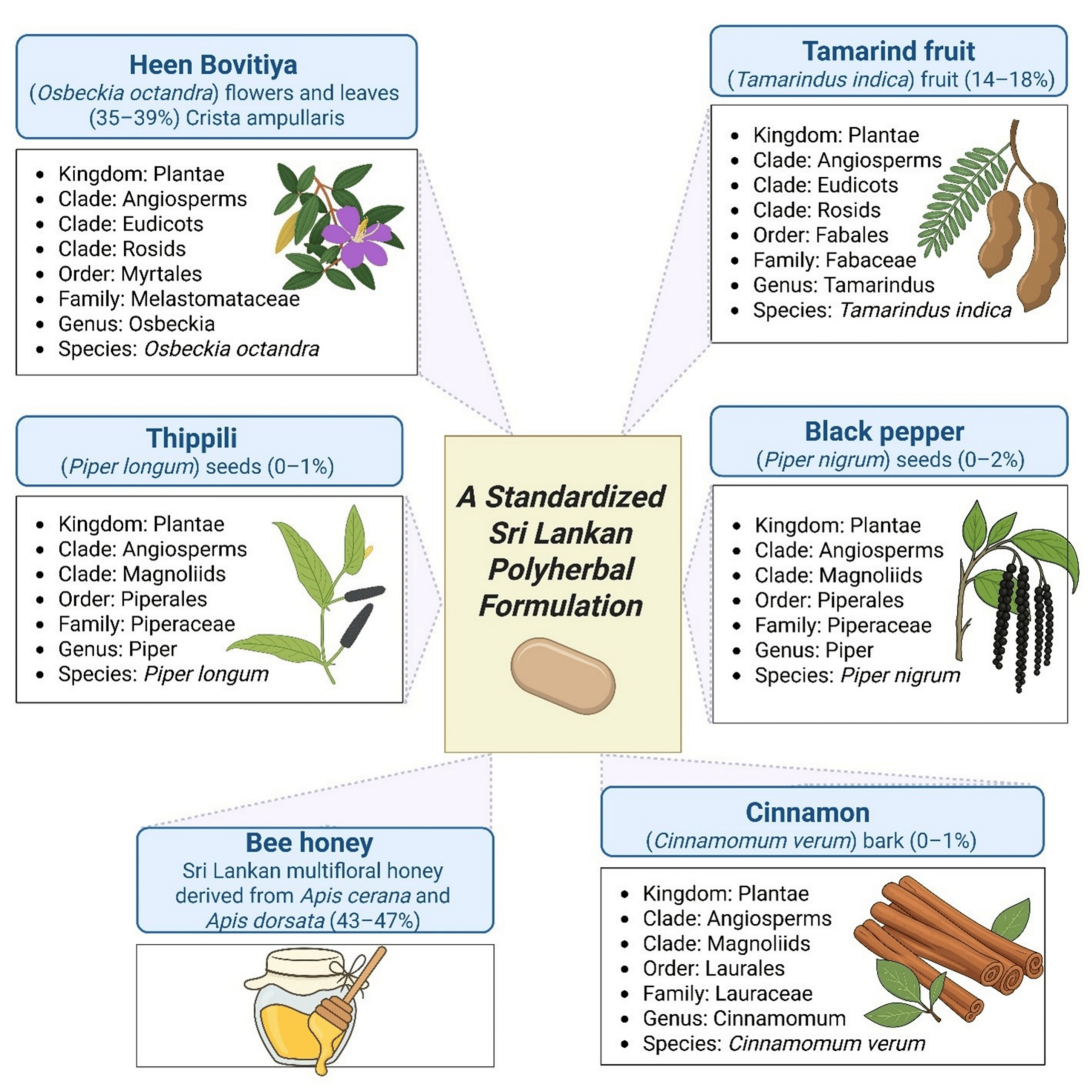
Overview of a Sri Lankan-origin standardized polyherbal formulation Image Credit: Kalmee P. Kariyawasam (author)

The term ‘standardized polyherbal formulation’ refers to a fixed-ratio combination of botanicals prepared under controlled manufacturing and quality assurance processes to ensure consistent chemical and therapeutic properties. These polyherbal formulation has been developed in Sri Lanka based on traditional Ayurvedic knowledge and supported by scientific evidence from the literature. As depicted in Figure 1, such formulations typically consist of "Heen Bovitiya" (*Osbeckia octandra*) 35-39%, tamarind fruit (*Tamarindus indica*) 14-18%, black pepper (*Piper nigrum*) 0-2%, long pepper or "thippili" (*Piper longum*) 0-1%, cinnamon (*Cinnamomum verum*) 0-1%, and bee honey 43-47%. This percentage of ingredients has been reported as safe for human consumption through literature reviews [8-12].

Each of these ingredients has been individually reported to exhibit hepatoprotective properties. For instance, *O. octandr*a has demonstrated antioxidant and anti-inflammatory effects in animal models of liver injury [7,13], while *T. indica* has shown hepatoprotection through modulation of oxidative stress markers and liver enzymes [7,14]. Although evidence derives from metabolic or antioxidant models, the underlying mechanisms are directly relevant to hepatocellular protection. Biospecies of *Piper*, such as *P. nigrum *and *P. longum*, are known for their bioactive alkaloids, which may contribute to hepatoprotection by inhibiting lipid peroxidation and promoting detoxification pathways [15]. *C. verum* exhibits antioxidant and anti-inflammatory properties that may support liver function [16], and bee honey has been associated with hepatoprotective and anti-fibrotic effects [14].

Although each of the ingredients demonstrates biochemical activity in isolation, no systematic synthesis of evidence has been performed to date to assess their combined effect. Despite the promising preclinical evidence for these individual ingredients, there is no clinical evidence evaluating the hepatoprotective efficacy of the full standardized polyherbal formulation as a whole. In Sri Lanka, based on Ayurvedic medicine, different commercially available products have been developed using this standardized polyherbal formulation (e.g., LivosBEE™), but systematic clinical evaluation remains absent. A systematic synthesis of the existing literature on its components is therefore essential to provide a rationale for future clinical testing.

Scoping reviews are particularly suited to this purpose, as they allow researchers to comprehensively map the available evidence, identify research gaps, and summarize the range and types of studies conducted in a field [17]. Unlike systematic reviews, which focus on specific outcomes, scoping reviews are broader in scope and can integrate findings from both preclinical and clinical studies to provide an overview of hepatoprotective potential. The primary aim of this scoping review is to identify and synthesize the available evidence on the hepatoprotective effects of the individual ingredients in the standardized Sri Lankan-origin polyherbal formulation, with particular emphasis on proposed mechanisms of action, types of studies conducted, and existing knowledge gaps. The findings will provide a scientific foundation for the formulation’s development, inform future preclinical and clinical research, and support evidence-based use of polyherbal interventions for liver health.

## Review

Methodology

This review followed the Preferred Reporting Items for Systematic Reviews and Meta-Analyses extension for Scoping Reviews (PRISMA-ScR) framework. The process comprised: (i) identifying the research question, (ii) defining eligibility criteria, (iii) systematically searching databases, (iv) selecting studies, and (v) charting and synthesizing data. The review also followed the methodological guidance outlined by Arksey and O’Malley, with refinements proposed by Levac et al. and further adapted through the Joanna Briggs Institute (JBI) guidelines for scoping reviews [18-20]. This framework offers a structured approach for systematically mapping available evidence, clarifying key concepts, and identifying research gaps.

Identifying the Research Question

**Figure 2:**
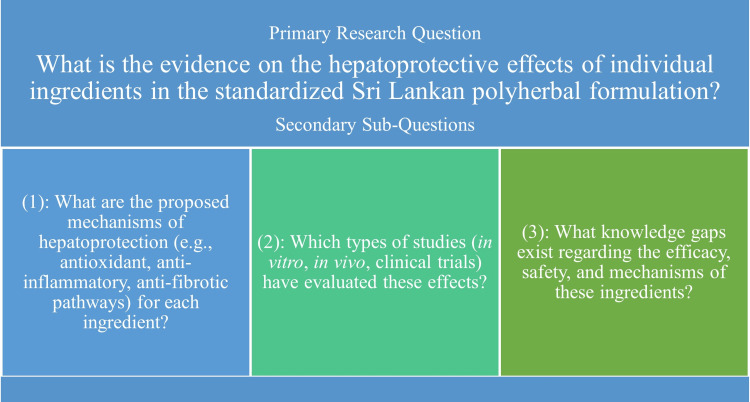
Overview of the primary and secondary research questions Image Credit: Kalmee P. Kariyawasam (author)

The primary research question guiding this review was to determine the evidence on the hepatoprotective effects of the individual ingredients in the standardized Sri Lankan polyherbal formulation (Figure 2). To address this, the review also explored several secondary questions. These included examining the proposed mechanisms underlying hepatoprotection, such as antioxidants, anti-inflammatory, and anti-fibrotic pathways. In addition, the review assessed the types of studies conducted, ranging from in vitro and in vivo investigations to clinical trials that have evaluated these effects. Finally, the review sought to identify existing knowledge gaps related to the efficacy, safety, and mechanisms of action of these ingredients, thereby providing direction for future research.

Eligibility Criteria

This review included studies involving preclinical models (e.g., cell lines and rodent studies) as well as human participants. Eligible studies were assessed for hepatoprotective effects, such as improvements in liver function, antioxidant activity, or anti-inflammatory effects. No restrictions were applied regarding geographic location or year of publication. Experimental studies, clinical trials, observational studies, and in vitro mechanistic studies were considered for inclusion. Studies were excluded if they did not specifically evaluate liver-related outcomes, were not published in English, or were unrelated to the ingredients of interest. Narrative reviews, editorials, and commentaries were excluded; these may have been used to identify primary studies, but were not included in data extraction.

Information Sources and Search Strategy

A comprehensive search was conducted across multiple electronic databases, including PubMed, Scopus, Web of Science, and Google Scholar, to identify relevant literature. Grey literature sources, such as conference proceedings, theses, and clinical trial registries, were also searched to capture unpublished or ongoing studies. Search terms were developed for each ingredient in LivosBEE™, *"Osbeckia octandra", "Tamarindus indica", "Piper nigrum", "Piper longum", "Cinnamomum verum", *and "bee honey", combined with hepatoprotection-related keywords, including “hepatoprotective”, “liver protection”, “liver injury”, “antioxidant”, and “anti-inflammatory”. Boolean operators (AND/OR) and Medical Subject Headings (MeSH) terms were applied where appropriate, and no restrictions were applied on publication date to ensure comprehensive coverage of all relevant evidence*.*

Study Selection, Data Extraction, and Synthesis

All identified records were imported into EndNote (Clarivate Plc, London, United Kingdom) for reference management and duplicate removal. Data from eligible studies were extracted using a standardized charting form, capturing key information including author, year, country, study design and model, intervention details (ingredient, dosage, formulation, duration), outcomes assessed (hepatoprotective markers, liver enzymes, histopathology), proposed mechanisms of action, and key findings. Findings were synthesized descriptively to map mechanistic patterns and evidence gaps rather than to quantify pooled effects by two reviewers. Discrepancies were resolved through discussion or consultation with a third reviewer. Extracted data were cross-checked for accuracy and completeness. The results were collated and presented quantitatively and qualitatively. Quantitative summaries included the number of studies per ingredient, study type, and experimental model, while qualitative synthesis identified key themes such as hepatoprotective mechanisms, evidence strength, and knowledge gaps. Tables and figures were used to map each ingredient against its hepatoprotective evidence and mechanisms, providing a comprehensive overview of current research and highlighting areas for further investigation.

Formal risk-of-bias scoring was not undertaken, as this review followed the scoping review framework. However, methodological characteristics of each included study, such as design type, sample size, blinding, extract standardization, and completeness of outcome reporting, were noted to contextualize evidence strength. 

The primary aim was to comprehensively map the breadth and nature of available evidence rather than critically appraise the quality of individual studies or pool outcomes quantitatively. Given the heterogeneity of included studies, ranging from in vitro and in vivo experiments to limited clinical investigations, the application of a uniform critical appraisal tool was not feasible. Instead, methodological limitations and potential biases within the included studies were qualitatively considered and highlighted to provide context and guide interpretation of the findings.

Results

A total of studies covering six key ingredients of the formulation*, O. octandra,* *T. indica*, *P. nigrum, P. longum, C. verum*, and bee honey, were identified. The priority was given to the evidence derived from in vivo animal studies evaluating hepatoprotective effects against chemical or diet-induced liver injury, while a lesser number of in vitro investigations explored antioxidant and anti-inflammatory mechanisms at the cellular level. There are a few clinical studies available that primarily focused on *P. nigrum*, *C. verum*, and bee honey. 

**Figure 3:**
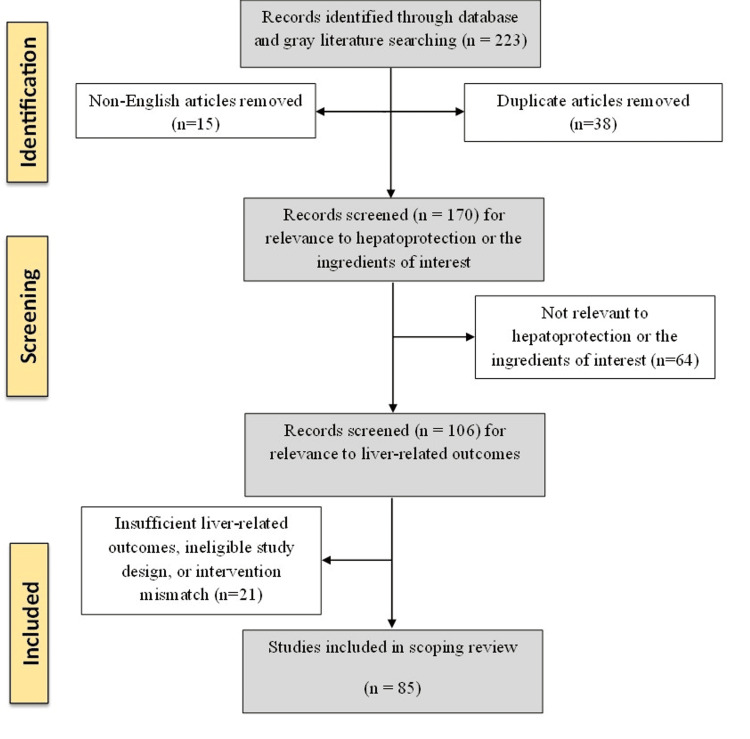
PRISMA-ScR flow diagram PRISMA-ScR: Preferred Reporting Items for Systematic reviews and Meta-Analyses extension for Scoping Reviews

A total of 223 articles were initially retrieved from electronic databases, including PubMed, Scopus, Web of Science, and Google Scholar, as well as grey literature sources. After removing 38 duplicate records, 15 non-English articles, and 64 studies deemed irrelevant to hepatoprotection or the ingredients of the standardized Sri Lankan-origin polyherbal formulation, 106 studies remained for assessment. Upon detailed evaluation, an additional 21 studies were excluded due to insufficient liver-related outcomes, ineligible study designs, or interventions that did not involve the ingredients of interest. Ultimately, 85 studies met the inclusion criteria and were incorporated into this scoping review. The study selection process is visually summarized in Figure 3, which presents the PRISMA-ScR flow diagram for the hepatoprotective effects of the ingredients that are used in the standardized polyherbal formulation.

The majority of included studies originated from South and Southeast Asia, particularly Sri Lanka, India, Pakistan, and Bangladesh, underscoring the ethnobotanical relevance of the formulation’s components. Preclinical investigations predominantly utilized rodent models such as Wistar rats, Sprague-Dawley rats, and Swiss albino mice, with liver injury induced by agents like carbon tetrachloride (CCl₄), thioacetamide, paracetamol, ethanol, and anti-tubercular drugs. A smaller subset of studies employed in vitro mechanistic assays or involved human clinical trials. Among the four major plant ingredients, *O. octandra *stood out with studies exclusively conducted in Sri Lanka, emphasizing its traditional use in Ayurveda and folk medicine for liver protection. In contrast, although *T. indica, P. nigrum, P. longum, and C. verum *are widely available in Sri Lanka, local research on their hepatoprotective properties was limited. Most Sri Lankan studies on these plants focused on other therapeutic areas such as antimicrobial, antioxidant, anti-diabetic, and anti-obesity effects, while international studies, though more numerous, often featured outdated findings, particularly for *T. indica, P. nigrum, and P. longum*. Notably, recent research has increasingly highlighted the hepatoprotective potential of *C. verum*.

Bee honey represents the only non-plant-based bioactive component in LivosBEE™, and the formulation exclusively utilizes Sri Lankan multifloral honey derived from *Apis cerana* and *Apis dorsata*. Accordingly, only studies investigating the hepatoprotective effects of honey from these two species, or closely related research, were considered eligible for inclusion. While Sri Lankan research on honey has largely centered on its antioxidant and antimicrobial properties, there remains a paucity of studies exploring its role in mitigating liver injury or other hepatic conditions. This gap highlights the need for further investigation into the therapeutic potential of *A. cerana *and *A. dorsata* honey in liver-related pathologies, especially given its inclusion as a key bioactive in LivosBEE™. The integration of both plant and non-plant components in the formulation reflects a holistic approach rooted in traditional medicine, warranting comprehensive scientific validation through robust experimental and clinical studies.

Hepatoprotective Profiles and Mechanistic Evidence of Individual Ingredients 

*O. octandra: O. octandra L. (DC.),* commonly known as Heen Bowitiya, is a small shrub endemic to Sri Lanka, belonging to the Melastomataceae family [21,22]. Traditionally, it has been used in Sri Lankan Ayurvedic medicine for the management of liver disorders, diabetes, jaundice, ascites, hemorrhoids, and hyperlipidemia [23,13]. The leaves are the primary medicinal part and are commonly incorporated into preparations such as "Kolakenda", which is used to support liver function in patients with diabetes and hepatitis [22].

Although scientific investigations on *O. octandra* remain limited, existing in vitro and in vivo studies consistently indicate its hepatoprotective potential. Various leaf preparations, including crude leaf suspension, boiled leaf extract, sonicated leaf extract, and isolated compounds such as pedunculagin, casuarinin, and gallic acid, have demonstrated strong antioxidant activity, reducing oxidative stress markers, enhancing the activity of endogenous antioxidant enzymes, and preserving protein synthesis in hepatocytes [7,8,24-26]. In addition, *O. octandra* exhibits anti-inflammatory effects by suppressing pro-inflammatory cytokines, including tumor necrosis factor alpha and interleukin-6, and immunomodulatory activity through inhibition of the complement system and reduction of reactive oxygen species production in human polymorphonuclear leukocytes [8,24]. Preclinical studies using multiple liver injury models, including thioacetamide, CCl_4_, D-galactosamine, paracetamol, and high-fat diet-induced liver injury, demonstrated normalization of liver enzymes such as alanine transaminase, aspartate transaminase, alkaline phosphatase, and gamma-glutamyl transferase, improvement in hepatic architecture, reduction of necroinflammatory activity, and mitigation of fibrosis through anti-fibrotic and anti-angiogenic pathways [26-30]. Mechanistic studies further revealed that the hepatoprotective effects are mediated through antioxidant, anti-inflammatory, anti-fibrotic, anti-angiogenic, immunomodulatory, and membrane-stabilizing actions, including inhibition of transforming growth factor beta/SMAD signaling and reduction of alpha-smooth muscle actin, transforming growth factor beta1, and vascular endothelial growth factor receptor 2 [13]. Collectively, these findings support the ethnopharmacological use of *O. octandra *as a liver tonic and highlight its potential as a key ingredient in standardized polyherbal formulations such as LivosBEE™.

A comprehensive summary of preclinical and in vitro studies evaluating the hepatoprotective effects of *O. octandra* is provided in Table 1.

**Table 1:** Summary of preclinical, in vitro, and clinical studies on hepatoprotective effects of heen bowitiya (Osbeckia octandra) ALT: alanine aminotransferase; ALP: alkaline phosphatase; AST: aspartate aminotransferase; α-SMA: alpha-smooth muscle actin; BLE: boiled leaf extract; BW: body weight; CAT: catalase; CCl₄: carbon tetrachloride; CLS: crude leaf suspension; 2,6-diMeNAPQI: 2,6-dimethyl-N-acetyl-p-benzoquinone imine; DM: dry matter; DPPH – 2,2-diphenyl-1-picrylhydrazyl (radical scavenging assay); GGT: gamma-glutamyl transferase; GSH: glutathione; HFD: high-fat diet; HLE: hexane leaf extract; ICR mice: Institute of Cancer Research mice; LDH: lactate dehydrogenase; IL-6: interleukin-6; MASLD: metabolic dysfunction-associated steatotic liver disease; MLE: methanol leaf extract; PMNs: polymorphonuclear leukocytes; PPAR-α: peroxisome proliferator-activated receptor alpha; ROS: reactive oxygen species; SLE: sonicated leaf extract; SOD: superoxide dismutase; TB: total bilirubin; TBH: tert-butyl hydroperoxide; TEAC: trolox equivalent antioxidant capacity; TGF-β1: transforming growth factor beta 1; TNF-α: tumor necrosis factor alpha; VEGF-R2: vascular endothelial growth factor receptor 2

Study Type	Experimental Model	Intervention	Key Outcomes	Mechanistic Insights	Reference
In vitro + Preclinical (in vivo)	HFD-induced MASLD mouse model; DPPH and TEAC antioxidant assays; Vero cell cytotoxicity assay	Aqueous extract of leaves	Strong antioxidant activity, low cytotoxicity; reduced weight gain, ALT, cholesterol, and triglycerides; improved hepatic steatosis and histology	Antioxidant (DPPH, TEAC); anti-inflammatory (↓TNF-α, IL-6); lipid metabolism regulation via PPAR-α	[7]
Preclinical (in vivo and in vitro)	Liver fibrosis model; hepatic stellate cell line LX-2	Crude leaf suspension (CLS), boiled leaf extract (BLE), ellagitannins (pedunculagin, casuarinin, gallic acid)	BLE more effective than CLS; ellagitannins reduced fibrosis in LX-2 cells	Inhibition of TGF-β/SMAD pathway; ellagitannins identified as a key bioactive compound	[13]
Preclinical (in vivo)	Thioacetamide-induced cirrhosis (Wistar rats)	CLS, BLE, SLE, MLE, HLE	↓Liver weight, ALT, AST, TNF-α, α-SMA, TGF-β1, VEGF-R2; BLE showed strong anti-angiogenic effect	Antioxidant, anti-inflammatory, anti-fibrotic, anti-angiogenic	[29]
Preclinical (in vivo)	carbon tetrachloride (CCl₄)-induced non-alcoholic fatty liver disease (NAFLD) -Wistar rats	Osbeckia octandra capsule (16.67 mg/kg)	↓ALT, AST, GGT; normalization of enzymes; prevention and therapeutic benefit	Antioxidant, anti-inflammatory, enzyme normalization	[31]
Clinical (human trial)	30 diabetic patients, Ayurveda hospital	Leaf powder infusion (2 spoonful in 30 ml warm water, 2× daily, 1 month)	Significant ↓fasting blood glucose; no adverse hepatic effects	Hypoglycemic effect; safe for liver (no hepatotoxicity)	[32]
Preclinical (in vivo)	carbon tetrachloride (CCl₄)- and D-galactosamine-induced hepatotoxicity in ICR mice	Polyherbal LINK LIVECARE™ (14 plants including Osbeckia octandra)	Significant hepatoprotection; ↓AST, ALT, ALP, TB; histology: reduced necrosis	Antioxidant, anti-inflammatory, membrane-stabilizing	[29]
Preclinical (in vivo)	carbon tetrachloride (CCl₄)-induced chronic liver damage (ICR mice)	Leaf extract (0.5 g DM/kg BW)	Improved protein, albumin; preserved liver architecture; reduced necroinflammation	Antioxidant, anti-inflammatory; preserved histology	[30]
Preclinical (in vivo and in vitro)	Paracetamol-induced hepatotoxicity (mice); rat hepatocytes exposed to 2,6-diMeNAPQI	Leaf extract (330 mg/kg in vivo; 500 μg/ml in vitro)	↑GSH, ↓AST, improved histology, protection with delayed addition	Antioxidant, membrane-stabilizing, cytoprotective	[25]
Preclinical (in vitro)	Rat hepatocytes exposed to galactosamine or TBH	Aqueous extract (500 μg/ml)	Preserved protein synthesis, ↓LDH and AST release, ↓lipid peroxidation	Antioxidant, membrane-stabilizing, cytoprotective	[23]
Preclinical (in vitro)	Human complement system and PMNs stimulated with zymosan	Aqueous whole plant extract	Strong anticomplement activity; inhibition of ROS production	Immunomodulatory (complement inhibition, ↓ROS in PMNs)	[24]
Preclinical (in vivo)	carbon tetrachloride (CCl₄)-induced liver dysfunction (albino rats)	Leaf extract (Osbeckia octandra) and aerial part of Melothria maderaspatana	Normalized ALT, AST, ALP; preserved histology; comparable to (+)-3-cyanidanol	Antioxidant, membrane-stabilizing; preventive and therapeutic	[28]
Preclinical (in vivo)	carbon tetrachloride (CCl₄)-induced liver damage (albino rats)	Leaf extracts of Osbeckia octandra and Pavetta indica	Osbeckia octandra prevented enzyme elevation and preserved histology; stronger hepatotonic effects than Pavetta	Antioxidant, membrane-stabilizing; preventive and therapeutic	[27]

*T. indica: T. indica*, commonly known as tamarind or in Sinhala "siyambala", is a multipurpose tropical tree of the family Fabaceae, subfamily Caesalpinioideae. Native to tropical Africa and widely naturalized across South Asia, including India and Sri Lanka, it is valued for its nutritional, medicinal, and industrial uses [33,34]. While tamarind fruit is extensively used in Sri Lankan culinary applications, research on its hepatoprotective effects within the local context is limited. The tree’s bioactive profile includes polyphenols, flavonoids, tannins, essential oils, and organic acids, which contribute to its antioxidants, anti-inflammatory, antidiabetic, antimicrobial, and cardioprotective properties [35]. The fruit pulp contains tartaric and malic acids (6.21-12.15% tartaric acid), vitamin C (5.67-11.35 mg/100 g), and flavonoids, while seeds provide polysaccharides (xyloglucan), proteins, essential amino acids, and unsaturated fatty acids (oleic acid ~20.32%, linoleic acid ~54.17%) [35-37]. Leaves and bark are rich in flavonoids such as quercetin and kaempferol, tannins, saponins, and alkaloids, which confer anti-inflammatory, antimicrobial, and antioxidant effects [38,39].

Preclinical studies have consistently demonstrated the hepatoprotective potential of tamarind extracts across multiple experimental models. In vitro studies using human hepatoblastoma-derived hepatocellular carcinoma cell line G2 (HepG2 cells) showed that methanolic flower extracts (5 mg/mL) improved hepatocyte viability and reduced oxidative stress, with significant nitric oxide (72%), 2,2-diphenyl-1-picrylhydrazyl (76%), and 2,2'-azino-bis(3-ethylbenzothiazoline-6-sulfonic acid) (82%) radical scavenging activity, reflecting antioxidant-mediated cytoprotection and multitarget bioactivity combining antidiabetic, antioxidant, and hepatoprotective pathways [40]. In vivo, Sprague Dawley rats with isoniazid- and rifampicin-induced liver injury exhibited dose-dependent restoration of liver enzymes (alanine aminotransferase (ALT), aspartate aminotransferase (AST), alkaline phosphatase (ALP), lactate dehydrogenase (LDH)), serum bilirubin, cholesterol, total protein, and albumin levels upon treatment with ethanolic stem bark extracts (100-200 mg/kg), with histopathology confirming hepatocyte protection and preservation of liver architecture [41]. Wistar rat pups prenatally exposed to aluminum chloride showed improved liver biochemical markers (malondialdehyde, caspase-3, Tumor necrosis factor alpha (TNF-α), ALT, AST, ALP) and mitigated histopathological damage following ethyl acetate leaf fraction administration (400-800 mg/kg), mediated through anti-lipid peroxidative, antiapoptotic, anti-inflammatory, and membrane-stabilizing mechanisms [42]. Adult Wistar rats with mercuric chloride-induced hepatotoxicity treated with aqueous fruit extract (250-500 mg/kg) demonstrated normalization of liver weight, improvement of body weight, reduction of liver enzyme elevations, and mitigation of histological liver damage, indicative of free radical scavenging, hepatocyte membrane stabilization, and overall hepatoprotection [12,43].

Advanced formulations, such as tamarind trypsin inhibitor (TTI) nanoencapsulated in chitosan-whey protein nanoparticles (ECW, 12.5 mg/kg), administered to adult Wistar rats on a high glycemic index diet, reduced blood glucose by 17%, AST by 39%, ALP by 24%, improved fibrosis markers (AST to Platelet Ratio Index and Fibrosis-4 Index), and restored liver morphology, highlighting antioxidant, anti-inflammatory, and hepatocyte-protective effects [44]. In hypertriglyceridemic Wistar rats, combinations of *T. indica *and *Murraya paniculata* leaf extracts (optimal ratio 350:50 mg/kg) significantly lowered triglycerides, reduced body weight gain, enhanced muscle lipoprotein lipase activity, and exhibited hepatoprotective effects via increased catalase activity and decreased malondialdehyde levels, demonstrating lipid-lowering and antioxidant-mediated liver protection [45]. Alloxan-induced diabetic rats treated with ethanolic fruit pulp extracts (300-500 mg/kg) showed significant reductions in serum glucose, improved lipid profile, normalized liver enzyme levels, and hepatocyte regeneration in a dose-dependent manner, with complete liver regeneration observed at 500 mg/kg [46]. Tamarind seed coat extracts (45-180 mg/kg) attenuated high-fat diet-induced nonalcoholic fatty liver disease in albino Wistar rats, reducing hepatomegaly, hepatic lipid accumulation, lipid peroxides, serum ALT, free fatty acids, micro- and macrosteatosis, body weight, and adiposity while improving insulin sensitivity [47]. Dietary incorporation of tamarind seeds (2-8%) in Sprague Dawley and spontaneously hypertensive rats lowered serum cholesterol and glucose and enhanced liver glycogen storage, indicating modulation of lipid and carbohydrate metabolism and potential insulin-sensitizing effects [48].

Further, in hypercholesterolemic hamsters, tamarind fruit pulp extract (5% in drinking water) significantly reduced serum triglycerides, total cholesterol, and low-density lipoprotein (LDL) cholesterol, increased high-density lipoprotein (HDL) cholesterol, enhanced hepatic antioxidant enzymes, and reduced hepatic lipid peroxidation. Gene expression analysis revealed upregulation of apolipoprotein A1, ATP-binding cassette g transporter 5, and LDL receptor genes and downregulation of 3-hydroxy-3-methylglutaryl-coenzyme A reductase and Mtp, suggesting mechanisms including enhanced cholesterol efflux, LDL cholesterol clearance, inhibition of cholesterol biosynthesis, and antioxidative protection [49]. Clinical studies in human volunteers (n=30) demonstrated that 15 mg/kg of dried tamarind fruit pulp significantly reduced total cholesterol and LDL cholesterol and decreased diastolic blood pressure, indirectly supporting liver health by reducing lipid burden and oxidative stress [50].

A summary of the studies on the hepatoprotective effects of *T. indica* and their findings is given in Table 2.

**Table 2:** Summary of preclinical, in vitro, and clinical studies on hepatoprotective effects of Tamarindus indica ALP: alkaline phosphatase; ALT: alanine aminotransferase; AST: aspartate aminotransferase; APRI: AST to Platelet Ratio Index; ABTS: 2,2'-azino-bis(3-ethylbenzothiazoline-6-sulfonic acid) radical scavenging assay; BP: blood pressure; CAT: catalase; CW: control nanoparticles without TTI; CVD: cardiovascular disease; DPPH: 2,2-diphenyl-1-picrylhydrazyl (radical scavenging assay); ECW: TTI nanoencapsulated in chitosan–whey protein nanoparticles; EFTI: ethyl acetate fraction of leaves;  ETS: ethanolic seed coat extract; GPx: glutathione peroxidase; HepG2: human hepatoblastoma-derived hepatocellular carcinoma cell line G2; HDL: high density lipoprotine; HFD: high-fat diet; HGLI: high glycemic index liver injury diet; HMG-CoA reductase: 3-hydroxy-3-methylglutaryl-coenzyme A reductase; LDH: lactate dehydrogenase; LDL-C: low-density lipoprotein cholesterol; LPL: lipoprotein lipase; MDA: malondialdehyde; MPE: Murraya paniculata extract; NO: nitric oxide; SD rats: Sprague Dawley rats; SHR: spontaneous hypertensive rats; SOD: superoxide dismutase; TBARS: thiobarbituric acid reactive substances; TIE: Tamarindus indica extract; TNF-α: tumor necrosis factor alpha

Study Type	Experimental Model	Intervention	Key Outcomes	Mechanistic Insights	Reference
Preclinical (in vitro)	HepG2 liver cells; antioxidant assays (NO, DPPH, ABTS)	Methanolic crude flower extract (5 mg/mL)	Improved cell viability; reduced oxidative stress; NO scavenging 72%, DPPH 76%, ABTS 82%	Free radical scavenging; antioxidant-mediated hepatoprotection; cytoprotective effects; multitarget bioactivity (antidiabetic, antioxidant, hepatoprotective)	[40]
Preclinical (in vivo)	Adult male Wistar rats; mercuric chloride-induced liver toxicity	Aqueous fruit extract (250 and 500 mg/kg)	Improved body weight; normalized liver weight; ↓ liver enzymes; mitigated histological damage	Antioxidant (free radical scavenging); membrane stabilization; liver enzyme normalization; hepatocyte protection	[12]
Preclinical (in vivo)	Hypertriglyceridemic Wistar rats; high-fat sucrose diet for 75 days	Combination of Tamarindus indica extract (TIE) and Murraya paniculata extract (MPE), 400 mg/kg/day; ratios 50:350 to 350:50	350:50 ratio most effective in lowering triglycerides; dose-dependent ↓ body weight; hepatoprotective effects in all combinations	Antioxidant activity (↑catalase, ↓MDA); enhanced muscle LPL activity; improved lipid metabolism	[45]
Preclinical (in vivo)	Wistar rat pups; prenatal aluminum chloride exposure	Ethyl acetate fraction of leaves (EFTI, 400 and 800 mg/kg); Vitamin E 300 mg/kg	Dose-dependent improvement in liver biomarkers (MDA, caspase-3, TNF-α, ALT, AST, ALP); mitigated histopathological damage	Anti-lipid peroxidative; antiapoptotic; anti-inflammatory; membrane-stabilizing; preserved liver architecture	[42]
Preclinical (in vivo)	Sprague Dawley rats; hepatic damage induced by isoniazid + rifampicin	Ethanolic stem bark extract (100 and 200 mg/kg)	Dose-dependent restoration of ALT, AST, ALP, LDH, bilirubin, cholesterol, total protein, albumin; histology confirmed protection	Membrane stabilization; improved liver metabolic and synthetic function; antioxidant and cytoprotective effects; preserved liver architecture	[41]
Preclinical (in vivo)	SD rats and Spontaneous Hypertensive Rats (SHR); basal diet ± tamarind seeds	Tamarind seeds incorporated at 2%, 4%, 8%	↓ Serum cholesterol (SD rats); ↓ glucose (SHR); ↑ liver glycogen storage	Modulation of lipid metabolism; enhanced glucose uptake and glycogen storage; insulin-sensitizing	[48]
Preclinical (in vivo)	Albino Wistar rats; HFD-induced non-alcoholic fatty liver disease	Seed coat extract (ETS, 45, 90, 180 mg/kg)	Attenuated hepatomegaly, hepatic lipid accumulation and lipid peroxides; ↓ALT, free fatty acids; reduced steatosis; ↓ body weight; improved insulin resistance	Antioxidant; anti-obesity; insulin sensitization; reduced hepatic lipid peroxidation	[47]
Preclinical (in vivo)	Alloxan-induced diabetic rats	Ethanolic fruit pulp extract (300 and 500 mg/kg); Metformin 150 mg/kg	Dose-dependent ↓ serum glucose; improved lipid profile; 500 mg/kg: complete liver regeneration; 300 mg/kg: partial improvement	Antioxidant activity; normalization of liver enzymes; hepatocyte regeneration	[46]
Preclinical (in vivo)	Hypercholesterolaemic hamsters; high-cholesterol diet for 10 weeks	Fruit pulp extract (dose not specified)	↓ serum triglycerides, total cholesterol, LDL-C; ↑ hepatic antioxidant enzymes; reduced hepatic lipid peroxidation	Enhanced cholesterol clearance (↑ApoA1, Abcg5, LDLR); ↓ cholesterol synthesis and triglyceride transport (↓HMG-CoA reductase, Mtp); antioxidative protection	[49]
Clinical in vivo	Human volunteers (n = 30; 25–49 y)	Dried and pulverized fruit pulp, 15 mg/kg	↓ Total cholesterol; ↓ LDL-C; ↓ diastolic BP; no effect on systolic BP or body weight	Lowering cholesterol reduces liver burden; antioxidant effects protect liver and support vascular health	[50]
Preclinical (in vivo and in vitro)	Hypercholesterolaemic hamsters; chow or atherogenic diet for 10 weeks	Fruit pulp extract (5% in drinking water)	↓ Total cholesterol 50%; ↓ non-HDL 73%; ↓ triglycerides 60%; ↑ HDL 61%; antioxidant activity (DPPH, superoxide, TBARS); ↑ antioxidant enzymes (SOD, CAT, GPx)	Reduces lipid accumulation in liver; normalizes liver enzymes; antioxidant protection; improved lipid metabolism; mitigates non-alcoholic fatty liver disease and CVD risk	[51]

Collectively, *T. indica* demonstrates hepatoprotective potential through multiple integrated mechanisms, including antioxidant activity, anti-inflammatory and antiapoptotic effects, modulation of lipid and glucose metabolism, membrane stabilization, normalization of liver enzymes, and preservation of hepatocyte architecture. Despite its extensive culinary use in Sri Lanka, research on direct liver protection is limited, with most evidence derived from preclinical in vivo and in vitro studies, highlighting the need for targeted clinical investigations to confirm hepatoprotective efficacy in the Sri Lankan context.

*P. nigrum: P. nigrum L.,* commonly known as black pepper, is renowned as the “King of Spices” not only for its culinary significance but also for its extensive pharmacological potential [52]. Its principal bioactive compound, piperine, along with other constituents such as piperic acid, piperlonguminine, pellitorine, piperolein B, piperamide, piperettine, and (-)-kusunokinin, has been widely studied for hepatoprotective, antioxidant, hypolipidemic, anti-inflammatory, and metabolic regulatory effects [53]. Preclinical investigations consistently demonstrate that *P. nigrum* extracts and piperine confer significant liver protection across diverse models of hepatotoxicity. In chemical-induced hepatotoxicity models, including CCl₄ and tert-butyl hydroperoxide, black pepper essential oil (BPEO) and piperine restored liver function markers such as ALT, AST, ALP, and bilirubin, suppressed lipid peroxidation (malondialdehyde, thiobarbituric acid reactive substances), and enhanced enzymatic antioxidants, including catalase, superoxide dismutase, and glutathione [52,54]. Histological analyses confirmed preservation of hepatic and renal tissue architecture, while piperine, though slightly less potent than silymarin, demonstrated a complementary hepatoprotective potential [55].

Drug-induced hepatotoxicity models, such as ethionamide and para-aminosalicylic acid in Sprague-Dawley rats, showed marked elevation in liver enzymes, bilirubin, and cholesterol, alongside disrupted histology, all of which were significantly ameliorated by co-administration of ethanolic extracts of Piper nigrum, suggesting antioxidant and cytoprotective mechanisms [55]. Dexamethasone-induced liver injury was mitigated by black pepper essential oil through modulation of peroxisome proliferator-activated receptor gamma coactivator 1-alpha/peroxisome proliferator-activated receptor alpha signaling, normalizing liver enzymes and reducing fibrosis and hepatocyte degeneration [56], while acetaminophen-induced oxidative stress and hepatotoxicity were alleviated by piperine via reductions in TNF-α, liver enzymes, and lipid peroxidation with restoration of enzymatic and non-enzymatic antioxidants [57]. Diet-induced metabolic stress models, including high-fat diet-induced obesity, revealed normalization of body weight, fat percentage, plasma glucose, lipid profile, leptin, adiponectin, and liver enzymes following *P. nigrum* treatment, highlighting both hepatoprotective and metabolic regulatory effects [58]. Sodium oxalate-induced oxidative stress models similarly demonstrated dose-dependent reductions in malondialdehyde, AST, ALT, and elevated serum vitamin C and catalase activity with *P. nigrum *administration [59]. Piperine also enhances the efficacy of other hepatoprotective agents, such as curcumin and *Aegle marmelos*, allowing lower doses to achieve significant protection in CCl₄ and paracetamol-induced hepatotoxicity, reflecting bioavailability enhancement and potentiation of antioxidant and anti-inflammatory activities [60,61].

The hepatoprotective effects of *P. nigrum* are primarily attributed to its antioxidant and anti-inflammatory properties. Piperine and black pepper extracts reduce oxidative stress markers such as thiobarbituric acid reactive substances, malondialdehyde, and conjugated dienes while enhancing enzymatic antioxidants (superoxide dismutase, catalase, glutathione peroxidase, glutathione S-transferase) and preserving glutathione [62]. Anti-inflammatory mechanisms include downregulation of TNF-α and IL-10, reduced leukocyte infiltration, and modulation of peroxisome proliferator-activated receptor gamma coactivator 1-alpha/peroxisome proliferator-activated receptor alpha signaling, maintaining hepatocyte integrity and preventing fibrosis. Piperine also exerts hypolipidemic and metabolic effects by lowering total cholesterol, triglycerides, LDL, and very LDL, increasing HDL, enhancing bile acid excretion, inhibiting 3-hydroxy-3-methylglutaryl-coenzyme A reductase, and modulating cholesterol transporters NPC1L1 and SR-BI [63-65]. It further improves thyroid hormones, testosterone, and apolipoprotein profiles. Beyond liver protection, *P. nigrum* exhibits antimicrobial, anticancer, anti-diabetic, neuroprotective, and analgesic activities [53]. Clinically, piperine supplementation in NAFLD patients improved lipid profiles, supporting its translational relevance [65].

A summary of the studies on the hepatoprotective effects of *P. nigrum* and their findings is given in Table 3.

**Table 3:** Summary of preclinical, in vitro, and clinical studies on hepatoprotective effects of Piper nigrum ALP: alkaline phosphatase; ALT: alanine aminotransferase; AST: aspartate aminotransferase; AP: alkaline phosphatase; Balb/c Mice: Bagg Albino/c mice; BPEO: black pepper essential oil; CAT: catalase; Caco-2: colorectal adenocarcinoma cells (human); CCl₄: carbon tetrachloride; ConA: concanavalin A; ETH: ethionamide; GSH: glutathione; GPx: glutathione peroxidase; GST: glutathione S-transferase; GR: glutathione reductase; HDL: high density lipoprotine; HFD: high-fat diet; HMG-CoA reductase: 3-hydroxy-3-methylglutaryl-coenzyme A reductase; IL-10: interleukin-10; LCAT: lecithin–cholesterol acyltransferase; LDL: low-density lipoprotein; LPL: lipoprotein lipase; MDA: malondialdehyde; PGC-1α: peroxisome proliferator-activated receptor gamma coactivator 1-alpha; NPC1L1: Niemann-Pick C1-Like 1 cholesterol transporter; PPAR-α: peroxisome proliferator-activated receptor alpha; PAS: para-amino salicylic acid; SOD: superoxide dismutase; SR-BI: scavenger receptor class B type I; TBARS: thiobarbituric acid reactive substances; TBIL: total bilirubin; t-BHP: tert-butyl hydroperoxide; TLE: tenofovir/lamivudine/efavirenz regimen; TNF-α: tumor necrosis factor alpha; VLDL: very low-density lipoprotein

Study Type	Experimental Model	Intervention	Key Outcomes	Mechanistic Insights	Reference
Clinical (randomized, double-blind, placebo-controlled)	Human subjects with non-alcoholic fatty liver disease (n = 170)	Piperine supplementation via black pepper powder, 12 weeks; placebo control	↓ Total cholesterol 15.13%, triglycerides 44.94%, LDL 21.58%; ↑ HDL 3.51%	Improves lipid metabolism; enhances lipid clearance; reduces hepatic fat accumulation; potential non-alcoholic fatty liver disease and cardiovascular risk reduction	[65]
Preclinical (in vivo)	Wistar rats; TLE-induced hepatotoxicity and dyslipidemia	Hydroethanolic extract of Piper nigrum stem (200, 400, 800 mg/kg, 28 days)	↓ ALP and triglycerides; ↑ catalase and GSH; normal liver/kidney histology; moderate weight reduction	Antioxidant enhancement; hepatoprotective; anti-inflammatory; metabolic regulation	[62]
Preclinical (in vivo)	Male Wistar rats; dexamethasone-induced hepatic injury	Black pepper essential oil (0.5 and 1 mL/kg); metformin 50 mg/kg	↓ ALT/AST; reduced hepatocyte degeneration, fibrosis, monocyte infiltration; improved liver morphology	Biochemical normalization; histopathological protection; antioxidant/anti-inflammatory; molecular modulation (↑ PPAR-α, ↓ PGC-1α)	[56]
Preclinical (in vivo)	Male Wistar rats; HFD (coconut oil + cholesterol + bile salts)	Black pepper (0.25 and 0.5 g/kg) or piperine 0.02 g/kg	↓ TBARS and conjugated dienes; restored SOD, CAT, GPx, GST; maintained GSH; systemic protection	Lipid peroxidation suppression; antioxidant enzyme preservation; glutathione maintenance; multi-organ protection	[62]
Preclinical (in vivo and in vitro)	Mice; carbon tetrachloride (CCl₄)-induced hepatotoxicity; Aspergillus flavus cultures	Black pepper essential oil (BPEO)	↑ Hepatic and renal antioxidant enzymes; normalized ALT, AST, ALP, TBIL; inhibited fungal growth; preserved liver/kidney tissue	Antioxidant; hepatoprotective; membrane stabilization; antifungal	[52]
Preclinical (in vivo)	Balb/c mice; conA-induced acute liver injury	70% methanolic extract of Piper nigrum (100–400 mg/kg)	Dose-dependent ↓ liver enzymes; improved oxidative stress markers; histology amelioration	Antioxidant enhancement; enzyme normalization; histopathological protection; dose-dependent efficacy	[66]
Preclinical (in vivo)	Wistar rats; ETH + PAS-induced hepatotoxicity	Ethanolic extract of fresh Piper nigrum seeds, 28 days	↓ ALT, AST, ALP, bilirubin, cholesterol, triglycerides; restored liver histoarchitecture	Hepatoprotective via enzyme stabilization, lipid metabolism improvement, antioxidant and cytoprotective effects	[55]
Preclinical (in vivo)	Wistar rats; carbon tetrachloride (CCl₄)-induced hepatotoxicity	Piperine + Aegle marmelos extract (25 and 50 mg/kg)	↓ ALT, AST, ALP, bilirubin, TNF-α, IL-10; restored GSH, SOD, CAT, GPx, GR, GST; liver protection	Antioxidant and anti-inflammatory; piperine enhances bioavailability and efficacy	[61]
Preclinical (in vivo)	Sprague–Dawley rats; HFD-induced obesity/metabolic disturbances	Piper nigrum extracts (hexane, ethyl acetate, ethanol, aqueous, 200 mg/kg, 42 days)	↓ Body weight, fat%; normalized glucose, lipid profile, liver enzymes; ↑ adiponectin, ↓ leptin; ↓ TBARS	Lipid metabolism modulation; hepatoprotective; antioxidant; metabolic/hormonal regulation	[58]
Preclinical (in vitro)	Caco-2 intestinal epithelial cells	Black pepper extract and piperine	↓ Cholesterol uptake; internalization of NPC1L1 and SR-BI; micelle solubility largely unchanged	Piperine reduces intestinal cholesterol absorption via transporter modulation	[64]
Preclinical (in vivo)	Wistar rats; paracetamol-induced hepatotoxicity	Combination extract: Curcumin + Piperine + Quercetin (CPQ), 7 days; silymarin reference	↑ Antioxidant activity (~50%); ↓ ALT, AST, ALP; superior hepatoprotection vs curcumin alone	Piperine enhances bioavailability; antioxidant and hepatoprotective synergy	[60]
Pre-clinical experimental	Hamsters; atherogenic diet	Piper species (Piper nigrum, Piper guineense, Piper umbellatum), 1 and 0.25 g/kg, 12 weeks	↓ Lipid profile alterations; restored antioxidant enzymes in the heart, liver, and kidney	Antioxidant protection; mitigates diet-induced oxidative stress; broad hepatorenal protection	[67]
Preclinical (in vivo)	Sprague-Dawley rats; acetaminophen hepatotoxicity	Piperine 25 mg/kg i.p.; silymarin 25 mg/kg	↓ AST, ALT, ALP, TNF-α; ↓ lipid peroxidation; restored antioxidant enzymes	Anti-oxidative protection; anti-inflammatory; hepatoprotection comparable to silymarin	[57]
Experimental in vivo	Rats; sodium oxalate–induced oxidative stress	Methanolic extract (MPN) up to 200 mg/kg, 10–20 days	↓ MDA; ↓ ALT/AST; ↑ catalase and vitamin C	Antioxidant; enzyme regulation; hepatoprotection	[59]
Preclinical (in vivo)	Male Wistar rats; high-fat diet–induced hypercholesterolemia	Piperine supplementation 40 mg/kg, 10 weeks	↓ Plasma total cholesterol, LDL, VLDL; ↓ HMG-CoA reductase; ↑ LPL, LCAT; ↑ bile acid and sterol excretion	Lowers circulating lipids; enhances cholesterol clearance; modulates lipid metabolism enzymes	[63]
Preclinical (in vivo and in vitro)	Hepatotoxicity induced by t-BHP and carbon tetrachloride (CCl₄) in mice	Piperine (alkaloid from Piper nigrum) vs silymarin	↓ Lipid peroxidation; prevented ALT/AP leakage; preserved GSH/thiols; hepatoprotection	Antioxidant defense; membrane stabilization; glutathione preservation; moderate hepatoprotection	[54]

Collectively, *P. nigrum* and piperine offer robust hepatoprotective, antioxidant, and hypolipidemic effects, mediated through enhancement of antioxidant defenses, anti-inflammatory signaling, membrane stabilization, and metabolic regulation. Synergistic interactions with other hepatoprotective agents further underscore their therapeutic potential. While Sri Lankan studies have primarily focused on antimicrobial and antioxidant effects, the extensive preclinical and emerging clinical evidence support *P. nigrum* as a promising hepatoprotective and metabolic-modulating agent, warranting further investigation in dietary and medicinal contexts.

*P. longum: P. longum L. (“Thippili”) *in the traditional medicine of Sri Lanka, commonly known as long pepper, is a perennial herb of the family Piperaceae, widely distributed in Sri Lanka, India, Malaysia, and the Philippines [68,69]. Traditionally, it has been used in Ayurvedic medicine for its stimulant, carminative, analgesic, and anti-inflammatory properties, particularly for the treatment of respiratory and digestive ailments [68,70]. The fruits of *P. longum *contain a diverse array of bioactive compounds, including alkaloids such as piperine, piperlongumine, piperlonguminine, and pipernonaline, along with newly identified constituents such as piperoic acid and guineesine [70-72]. Several of these compounds, particularly piperine and piperlongumine, have demonstrated notable hepatoprotective, antioxidant, and antihyperlipidemic activities in preclinical studies.

The hepatoprotective potential of *P. longum* has been extensively investigated in diverse experimental models of liver injury. In CCl₄-induced hepatotoxicity in rats, methanolic and ethanol extracts of *P. longum* were shown to significantly restore elevated serum liver enzymes (AST, ALT, ALP), reduce bilirubin levels, and improve antioxidant enzyme activities (superoxide dismutase, glutathione peroxidase, catalase), indicating attenuation of oxidative stress and stabilization of hepatocellular membranes [73,74]. Similarly, the traditional milk extract of *P. longum*, administered orally for 21 days, offered comparable protection against chronic CCl₄-induced liver damage, highlighting the efficacy of ethnomedicinal preparations [75]. The ethanol extracts also demonstrated antifibrotic activity by reducing hydroxyproline content and liver weight in CCl₄-induced fibrotic rats, suggesting inhibition of collagen deposition and suppression of fibrosis [73].

Modern derivatives of *P. longum*, such as piperlongumine and dihydroxy piperlongumine, have been evaluated in metabolic liver disorders. Piperlongumine administration in zebrafish and high-fat diet-induced mouse models of MAFLD significantly improved hyperglycemia, hepatic steatosis, and insulin resistance through mechanisms including inhibition of hepatic gluconeogenesis, suppression of de novo lipogenesis, and modulation of cAMP response element-binding protein and sterol regulatory element-binding protein 1c signaling pathways [72]. Dihydroxy piperlongumine, a demethylated derivative with strong antioxidant activity, effectively reduced hepatic lipid accumulation and oxidative stress in high-cholesterol diet-induced NAFLD in zebrafish, partially through upregulation of antioxidant and lipid-lowering genes [69]. These studies demonstrate the dual metabolic and hepatoprotective potential of bioactive alkaloids from *P. longum*.

Additional models of liver injury further support its hepatoprotective role. In ethanol-induced hepatotoxicity, methanolic extracts of *P. longum* significantly lowered serum biomarkers, improved antioxidant enzyme activities, and protected liver histology, comparable to the standard hepatoprotective drug silymarin. Co-administration of aqueous extracts or piperine with antitubercular drugs prevented oxidative stress-induced hepatocellular injury by restoring glutathione levels and reducing lipid peroxidation [71]. Moreover, extracts of *P. longum* demonstrated protective effects in aluminium chloride-induced hepatotoxicity, restoring liver enzymes and attenuating biochemical liver damage [76], and the root aqueous extract showed hepatoprotective, antihyperglycemic, and antihyperlipidemic effects in streptozotocin-induced diabetic rats, supporting organ-protective and metabolic benefits [77]. Interestingly, the anthelmintic effect of alcoholic extracts of *P. longum* against liver amphistomes (*Gigantocotyle explanatum*) may indirectly support its hepatoprotective role by preventing parasite-induced hepatic damage [78].

Taken together, preclinical studies provide compelling evidence that *P. longum* exerts hepatoprotective effects through antioxidant activity, inhibition of lipid peroxidation, cytoprotection of hepatocytes, antifibrotic activity, and modulation of metabolic pathways. These findings are summarized in Table 4, which outlines the experimental models, interventions, key outcomes, and mechanistic insights of *P. longum*’s hepatoprotective studies. While the plant is widely used in traditional medicine in Sri Lanka and other regions, human clinical trials remain lacking, highlighting the need for further translational research to validate its therapeutic potential. These studies consistently demonstrate antioxidant and anti-inflammatory activity, contributing to observed hepatoprotective effects across diverse experimental models.

**Table 4:** Summary of preclinical and in vitro studies on hepatoprotective effects of Piper longum AlCl₃: aluminum chloride; ALP; alkaline phosphatase; ALT: alanine aminotransferase; AST: aspartate aminotransferase; Ca²⁺: calcium ions; CAT: catalase; CCl₄: carbon tetrachloride; CREB: cAMP response element-binding protein; CRTC2: CREB-regulated transcription coactivator 2; DHPL: dihydroxy piperlongumine; GPx: glutathione peroxidase; HbA1c: glycated hemoglobin; HFD: high-fat diet; HCD: high-cholesterol diet; HDL: high-density lipoprotein; LDL: low-density lipoprotein; MASLD: metabolic dysfunction-associated steatotic liver disease; NAFLD: non-alcoholic fatty liver disease; PL: piperlongumine; ROS: reactive oxygen species; SOD: superoxide dismutase; SREBP-1c: sterol regulatory element-binding protein 1c; STZ: streptozotocin; TC: total cholesterol; TG: triglycerides; TP: thromboxane prostanoid; VLDL: very-low-density lipoprotein

Study Type	Experimental Model	Intervention	Key Outcomes	Mechanistic Insights	Reference
Experimental (in vivo and zebrafish)	Zebrafish and mouse model; HFD-induced MAFLD	Piperlongumine (PL)	↓ Hyperglycemia, hepatic steatosis, insulin resistance; ↓ hepatic gluconeogenesis; inhibition of de novo lipogenesis; improved insulin sensitivity	Hypoglycemic via CRTC2–CREB; lipid-lowering via ↓ SREBP-1c; anti–insulin resistance via TP/Ca²⁺ signaling; TP receptor as molecular target	[72]
Experimental (in vivo)	Zebrafish; HCD-induced non-alcoholic fatty liver disease (NAFLD)	Dihydroxy piperlongumine (DHPL)	↓ Hepatic lipid accumulation and triglycerides; improved liver histology; ↑ antioxidant activity; ↓ oxidative stress	Strong antioxidant; upregulation of antioxidant/lipid-lowering genes; downregulation of lipogenic genes; halted non-alcoholic fatty liver disease (NAFLD) progression	[69]
Experimental (in vivo + in vitro)	Wistar rats; ethanol-induced hepatotoxicity	Methanolic extract of Piper longum (100–400 mg/kg) vs silymarin	↓ AST, ALT, ALP, bilirubin; restored liver weight/volume; ↑ SOD, GPx, CAT; histological protection comparable to silymarin	Antioxidant defense (ROS scavenging); hepatoprotection via membrane stabilization and enzyme restoration	[74]
Preclinical (in vivo)	Charles Foster rats; carbon tetrachloride (CCl₄)- induced hepatotoxicity	LIV 52 (1 mL/kg, p.o.), ethanolic extract of Abutilon indicum + Piper longum (100–400 mg/kg), phytosomal formulation (100 mg/kg)	Combined extract and phytosomes showed significant hepatoprotection; histopathology confirmed improved liver integrity	Enhanced bioavailability (phytosomes); antioxidant action; liver enzyme normalization; histological protection; synergistic effect; cytoprotective mechanisms	[79]
Experimental (in vitro + in vivo)	Human cancer cell lines and AlCl₃-induced hepatotoxicity in rats	Multiple solvent extracts of Piper longum fruit	In vitro: 84–95% cytotoxicity; induced sub-G1 DNA fraction; antioxidant membrane protection (71–65%); in vivo: restored liver enzymes	Anticancer via apoptosis; antioxidant defense; hepatoprotection via free radical scavenging	[76]
Experimental (in vivo)	Male Wistar rats; STZ-induced diabetes	Aqueous root extract (PlrAqe), 200 mg/kg, short-term (6 h) and long-term (30 days)	↓ Fasting blood glucose, HbA1c; ↓ TC, TG, LDL, VLDL; ↑ HDL; ↓ liver/renal markers; no toxicity	Antidiabetic; antihyperlipidemic; hepatoprotective; renoprotective; antioxidant and cytoprotective	[77]
Experimental (in vivo)	Rats; antitubercular drug–induced hepatotoxicity	Aqueous extract of Piper longum and piperine	↑ Glutathione; ↓ lipid peroxidation; prevented oxidative stress; histology comparable to control	Antioxidant restoration; prevention of hepatocellular injury during anti-TB therapy	[71]
Experimental (in vivo)	Wistar rats; carbon tetrachloride (CCl₄) - induced chronic liver toxicity	Traditional milk extract of Piper longum (200 mg/day) vs silymarin	↓ AST, ALT, ALP, bilirubin; hepatocellular protection; efficacy comparable to silymarin	Antioxidant defense; hepatoprotective via potentiated bioactivity; validation of ethnomedicinal use	[73]
Experimental (in vitro, parasitological assay)	Gigantocotyle explanatum (liver amphistome)	Alcoholic extract of Piper longum	Induced paralysis within 15–20 min; irreversible; similar to Allium sativum	Anthelmintic effect via interference with neurotransmission/muscular contraction; indirectly supports hepatoprotective role by preventing parasite-induced liver damage	[78]
Experimental (in vivo)	Rats; carbon tetrachloride (CCl₄) -induced liver fibrosis	Ethanol extract of Piper longum fruits	↓ Hydroxyproline; ↓ serum liver enzymes; ↓ liver weight; clear antifibrotic activity	Collagen deposition inhibition; hepatoprotective via serum biomarker normalization; fibrosis suppression	[73]

*C. verum: *Cinnamon is obtained from the inner bark of trees belonging to the genus *Cinnamomum*. The two main varieties of cinnamon are *C. verum* (true cinnamon), previously known as *Cinnamomum zeylanicum*, and *Cinnamon cassia*, also referred to as *Cinnamomum aromaticum* or Chinese cinnamon [80]. Beyond its widespread culinary use, cinnamon has been traditionally employed in Ayurvedic medicine as a remedy for respiratory, digestive, and gynecological ailments. Different parts of the plant, including bark, leaves, and roots, contain similar classes of hydrocarbons in varying proportions, with cinnamaldehyde being the major component in the bark, eugenol in the leaves, and camphor in the roots [81].

*C. verum*, also called “Ceylon cinnamon” or “true cinnamon,” is indigenous to Sri Lanka and the southern parts of India. A major distinction between *C. verum *and* C. cassia *is their coumarin content. Coumarins are plant-derived compounds with anticoagulant, carcinogenic, and hepatotoxic properties [80-82]. While *C. cassia* contains high levels of coumarins, posing potential health risks with regular consumption, *C. verum* contains negligible amounts and is considered safe for long-term use [83]. In vitro and in vivo studies in both animals and humans have demonstrated multiple beneficial effects of *C. verum*, including anti-inflammatory, antimicrobial, cardioprotective, neuroprotective, and anti-cancer activities [84,85]. In animal models of diabetes, *C. verum* has been shown to attenuate weight loss, reduce fasting blood glucose, lower LDL, increase HDL cholesterol, decrease glycated hemoglobin, and enhance circulating insulin levels. Moreover, it exhibits protective effects against diabetic neuropathy and nephropathy [86].

The bioactive compounds cinnamaldehyde and eugenol appear to be key contributors to its antioxidant, anti-inflammatory, and anti-apoptotic properties. Of particular interest is its protective potential in both alcoholic and NAFLD. Experimental studies consistently demonstrate that cinnamon extracts, essential oils, and isolated compounds can reduce elevated liver enzyme levels and improve histopathology. For instance, pre-treatment with cinnamon bark essential oil in rats exposed to CCl_4_ significantly attenuated elevated serum ALT, AST, ALP, and Gamma-glutamyl transferase, restored antioxidant enzyme activities, including superoxide dismutase and catalase, reduced lipid peroxidation, and prevented histopathological lesions in liver tissues [81,83]. Similarly, administration of cinnamon ethanolic or hydroalcoholic extracts mitigated acetaminophen- and isoniazid-induced hepatotoxicity by restoring serum biomarkers, decreasing malondialdehyde levels, enhancing endogenous antioxidants, and modulating apoptotic gene expression [87,88]. These studies collectively indicate that *C. verum* not only stabilizes hepatic enzyme profiles and preserves tissue architecture but also counteracts oxidative stress and apoptosis in hepatocytes, supporting its potential therapeutic application in liver disorders.

Clinical evidence further supports the hepatoprotective potential of *C. verum*. In patients with NAFLD, oral supplementation with cinnamon alone or in combination with other herbal components such as *Melissa officinalis* and *Urtica dioica* resulted in significant reductions in ALT levels and improvements in sonographic fatty liver grades, indicating alleviation of hepatic fat accumulation and inflammation [89,90]. Mechanistically, these protective effects are largely attributed to antioxidant activity, reduction of reactive oxygen species, anti-inflammatory effects, stabilization of hepatocyte membranes, and inhibition of lipid peroxidation [91,92]. Moreover, co-administration with other bioactive compounds, such as ginger extracts, appears to provide synergistic hepatoprotective effects, enhancing antioxidant capacity and reducing oxidative liver injury more effectively than either extract alone [93].

*O*verall, the evidence from preclinical and clinical studies consistently demonstrates that *C. verum* exhibits potent hepatoprotective effects, largely driven by its antioxidant, anti-inflammatory, and anti-apoptotic activities, with key bioactive compounds such as cinnamaldehyde and eugenol playing central roles in mitigating liver injury and preserving hepatic function [85-87,90]. Table 5 summarizes the hepatoprotective effects of C. verum, highlighting its ability to confer significant liver protection through multiple mechanisms.

**Table 5:** Preclinical and in vitro studies on hepatoprotective effects of Cinnamomum verum 2-MCA: 2-methoxycinnamaldehyde; ALI: acute liver Injury; ALP: alkaline phosphatase; ALT: alanine aminotransferase; APAP: acetaminophen; AST: aspartate aminotransferase; Bad: Bcl-2-associated agonist of cell death; Bak: Bcl-2 homologous antagonist/killer; Balb/c mice: Bagg Albino/c mice; Bax: Bcl-2-associated X protein; Bcl-2: B-cell lymphoma 2; Bcl-XL: B-cell lymphoma-extra large; BID: *bis in die* (twice daily); CAT: catalase; CBO: cinnamon bark oil; CCl₄: carbon tetrachloride; COX-2: cyclooxygenase-2; CvEO: *Cinnamomum verum* essential oil; CYP2D1: cytochrome P450 2D1; EO: essential oil; FBS: fasting blood sugar; GC-MS: gas chromatography-mass spectrometry; GPx: glutathione peroxidase; γ-GT/GGT: gamma-glutamyl transferase; GSH: glutathione; Gy: Gray; HDL: high-density lipoprotein; Hep 3B cells: a human hepatocellular carcinoma (liver cancer) cell line; LDL-C: low-density lipoprotein cholesterol; LDL: low-density lipoprotein; LDH: lactate dehydrogenase; LFT: liver function test; MDA: malondialdehyde; NAFLD: non-alcoholic fatty liver disease; NF-κB: nuclear factor kappa-light-chain-enhancer of activated B cells; ns: not statistically significant; PGE2: prostaglandin E2; ROS: reactive oxygen species; SOD: superoxide dismutase; T2DM: type 2 diabetes mellitus; TC: total cholesterol; TBARS: thiobarbituric acid reactive substances; TG: triglycerides; TSB: total serum bilirubin

Study Type	Experimental Model	Intervention	Key Outcomes	Mechanistic Insights	Reference
Experimental in vivo (aquaculture)	Rainbow trout; 1–12 g/kg cinnamon powder for 60 days	Dietary cinnamon powder	↓ AST, ALT; ↑ SOD, CAT; ↓ MDA; ↑ protein, ↓ fat at high doses	Enhanced antioxidant defense; hepatoprotective; improved physiological and muscle composition	[94]
Randomized clinical trial	Adults with LDL-C 100–190 mg/dL (including T2DM)	1000 mg/day standardized Cinnamomum zeylanicum extract for 12 weeks	No significant LDL-C change; ↓ FBS, more pronounced in T2DM; safe	Improves glycemic control, likely via cinnamaldehyde; limited lipid-lowering effect	[85]
Single-blind clinical trial	Adults with non-alcoholic fatty liver disease	Cinnamomum zeylanicum capsules 500 mg BID, 60 days; control: vitamin E 400 mg BID	↓ fatty liver grade; improved dyspepsia, anorexia, malaise; minimal changes in LFTs; lipid profile unchanged	Antioxidant and anti-inflammatory hepatoprotective effects; comparable to vitamin E	90]
Experimental in vivo	Adult male rats; Cinnamomum verum EO analyzed via GC-MS	EO major compounds: cinnamaldehyde 46%, 9-Methoxybicyclo [6.1.0] nona-2,4,6-triene 31%, muurolene 7%, copaene 1.6%	Reduced histopathological abnormalities: hepatocyte swelling, necrosis, steatosis; preserved liver architecture	Antioxidant, anti-inflammatory; stabilizes hepatocyte structure; cytoprotection	[86]
Experimental in vivo	Male Wistar rats; isoniazid-induced hepatotoxicity	Cinnamon hydroalcoholic extract 50–200 mg/kg, 14 days	↓ biochemical liver markers, ROS, lipid peroxidation; improved histopathology	Antioxidant hepatoprotection; preserves liver tissue and function	[88]
Experimental in vivo	Wistar rats; carbon tetrachloride (CCl₄) -induced acute kidney injury	Cinnamon 25–50 mg/kg ± ginger 125–250 mg/kg hydroalcoholic extracts, 14 days	Restored renal biochemical markers and antioxidant enzymes; histology improved; combination more effective	Antioxidant and free radical scavenging; synergistic protection	[95]
Experimental in vivo	Balb/c mice; APAP-induced acute liver injury	Cinnamon extract 200 mg/kg, cinnamaldehyde 10 mg/kg, kaempferol 10 mg/kg, 14 days pretreatment	↓ serum liver biomarkers; improved histology; modulated apoptotic genes (↓ Bad, Bax, Caspase-3; ↑ Bcl-2); cinnamaldehyde and kaempferol more potent	Antioxidant activity; anti-apoptotic; potent bioactive components	[96]
Experimental in vivo	Male Wistar rats; type 1 and 2 diabetes	Cinnamon alone or ± insulin/metformin, 14 days	Modulated CYP2D1 activity; improved hepatic clearance; normalization of metabolism	Supports hepatic metabolic function and drug clearance in diabetes	[97]
Experimental in vivo	Rats; acetaminophen-induced hepatotoxicity	Cinnamon bark ethanolic extract 100 and 200 mg/kg; silymarin 100 mg/kg	↓ ALT, AST, ALP, bilirubin, total cholesterol; ↑ antioxidant enzymes; improved histology	Antioxidant activity; prevents lipid peroxidation; stabilizes hepatic membranes	[87]
Experimental in vivo	Male Wistar rats; carbon tetrachloride (CCl₄) -induced liver injury	Cinnamon 50 mg/kg ± ginger 125 mg/kg; silymarin 100 mg/kg, 14 days	↓ AST, ALT, ALP, bilirubin, MDA; restored antioxidant capacity and protein; combination more effective than individual extracts	Antioxidant-mediated hepatoprotection; synergistic effect of cinnamon + ginger	[93]
Experimental in vivo	Rats; carbon tetrachloride (CCl₄) -induced hepatic and renal toxicity	CvEO 70 or 100 mg/kg, 7 days prior to carbon tetrachloride (CCl₄)	↓ ALT, AST, ALP, γ-GT, LDH, TC, TG, LDL; ↑ HDL; ↓ TBARS and protein carbonyl; prevented histopathological lesions	Antioxidant activity; ROS quenching; lipid peroxidation prevention; cytoprotection in liver and kidney	[81]
Experimental in vivo	Rats; metanil yellow-induced hepatotoxicity	Cinnamaldehyde co-administration	Restored liver enzymes, bilirubin, creatinine; ↑ SOD, CAT, GSH; ↓ MDA; histology improved	Antioxidant and free radical scavenging; hepatoprotection	[92]
Experimental in vitro + in vivo	Hep 3B cells; nude mice xenografts	2-Methoxycinnamaldehyde (2-MCA)	Anti-proliferative; apoptosis induction (↑ Bax, Bak; ↓ Bcl-2, Bcl-XL); mitochondrial dysfunction; caspase activation; lysosomal vacuolation; NF-κB/COX-2/PGE2 suppression; topoisomerase inhibition; ↓ tumor growth in vivo	Multi-target activity: intrinsic apoptosis, anti-inflammatory, topoisomerase inhibition, lysosomal-mediated cell death; potential anticancer agent	[98]
Experimental in vivo	Male rats; paracetamol-induced hepatotoxicity	Cinnamon EO (hydrodistilled, major compounds: cinnamaldehyde 68%, eugenol 11%, others) 8 weeks; silymarin	↓ ALT, AST, ALP, total lipids, TG, TC, LDL-C, liver MDA; ↑ serum protein, albumin, HDL; improved liver antioxidant enzymes	Bioactive compounds reduce oxidative stress, inhibit lipid peroxidation, restore liver function	[99]
Experimental in vivo	Male albino rats; gamma irradiation 3 Gy	Cinnamon aqueous extract 200 mg/kg, 15 or 40 days	↓ hepatic and cardiac tissue injury, necrosis, apoptosis; better with 40-day treatment	Antioxidant bioactives (eugenol, cinnamic acid, cinnamaldehyde); membrane stabilization; inhibits apoptosis	[91]
Experimental in vivo	Rats; carbon tetrachloride (CCl₄) -induced liver injury	Cinnamon bark ethanolic extract 0.01–0.1 g/kg, 28 days	↓ AST, ALT, ALP; ↑ SOD, CAT; preserved histology	Antioxidant-mediated hepatoprotection; prevents oxidative stress–induced damage	[84]
Experimental in vivo	Male Wistar rats; carbon tetrachloride (CCl₄) -induced reproductive toxicity	Cinnamon bark oil 100 mg/kg, daily; carbon tetrachloride (CCl₄) weekly, 10 weeks	↑ reproductive organ weights; improved sperm quality, oxidative stress markers, apoptotic index; histology improved	Antioxidant activity; anti-apoptotic; protects reproductive organs	[100]
Before-after clinical trial	35 non-alcoholic fatty liver disease (NAFLD) patients	Herbal infusion: Melissa officinalis 1.5 g, Cinnamomum zeylanicum 0.5 g, Urtica dioica 0.25 g per 100 mL, twice daily, 30 days	↓ ALT; AST and ALP decreased (ns); improved sonographic fatty liver grade; age correlated with ALT/AST changes	Likely antioxidant and hepatoprotective effects; reduces hepatic inflammation and fat accumulation	[89]
Experimental in vivo	Rats; carbon tetrachloride (CCl₄) -induced oxidative stress	Cinnamon aqueous/ethanolic extracts 200 mg/kg, 7 days prior	↓ AST, ALT, MDA; ↑ SOD, CAT; ethanolic extract more potent; histology improved	Free radical scavenging via polyphenols; antioxidant-mediated hepatoprotection	[83]
Experimental in vivo	Rabbits; carbon tetrachloride (CCl₄) -induced ALI	Aqueous extracts of celery or cinnamon, 5 days post-carbon tetrachloride (CCl₄)	↓ ALP, AST, TSB; histology improved; celery slightly stronger	Mild hepatoprotection via antioxidant and membrane-stabilizing activity	[101]

Bee honey: Bee honey has emerged as a natural nutraceutical with significant hepatoprotective potential, largely due to its rich composition of bioactive compounds such as phenolics, flavonoids, vitamin C, carotenoids, and enzymatic antioxidants including glucose oxidase and catalase [14,102]. The antioxidant capacity of honey is closely linked to its total phenolic content and colour, with darker honeys generally exhibiting higher phenolic concentrations and greater radical scavenging activity [102]. In Sri Lanka, research has identified *A. cerana* and *A. dorsata* as the primary honey-producing species, producing both unifloral and multifloral honeys in regions such as Nuwara Eliya and Welimada [103]. Despite this diversity, studies on the hepatoprotective properties of locally produced honey are limited compared with international research on Manuka honey, indicating a clear gap in understanding the bio-efficacy of Sri Lankan varieties [103].

Preclinical studies consistently demonstrate that honey can mitigate liver injury induced by a range of hepatotoxic insults. For instance, *A. cerana* honey has been shown to reduce serum Alanine aminotransferase and Aspartate aminotransferase levels, suppress hepatic malondialdehyde accumulation, and enhance antioxidant enzyme activities in alcohol- and bromobenzene-induced liver injury models [104,105]. Similarly, bee bread supplementation alleviated high-fat diet-induced MAFLD by improving endogenous antioxidant defenses, decreasing oxidative stress markers, and attenuating inflammatory responses [106]. In models of cholestasis and CCl₄-induced hepatotoxicity, honey supplementation reduced hepatocellular damage markers, modulated nitric oxide metabolism, and strengthened systemic and hepatic antioxidant capacity [11,107]. Collectively, these studies illustrate that honey exerts hepatoprotection across diverse experimental models, supporting its potential applicability in multiple liver disorders.

Mechanistically, honey exerts its hepatoprotective effects through a combination of antioxidant, anti-inflammatory, and anti-fibrotic pathways. Its phenolic and flavonoid compounds, along with vitamins and enzymes, enhance enzymatic antioxidants such as superoxide dismutase, catalase, and glutathione peroxidase while increasing non-enzymatic defenses like thiols, thereby reducing oxidative stress and lipid peroxidation [104,107]. Honey also suppresses pro-inflammatory mediators, including TNF-α, nuclear factor kappa beta, and adenosine deaminase, and inhibits fibrogenic signaling through down-regulation of transforming growth factor beta 1 [11,104,105]. These multifaceted actions not only prevent hepatocyte injury but also reduce progression toward fibrosis and steatohepatitis, highlighting honey’s potential in both protective and therapeutic contexts.

Importantly, the hepatoprotective efficacy of honey is influenced by dosage, bioactive content, and phenolic richness. Moderate supplementation generally provides optimal protection, whereas excessive intake may induce metabolic stress or oxidative imbalance, as observed with higher doses of *A. dorsata* honey [106]. Additionally, honey samples with higher total phenolic content and total antioxidant capacity confer stronger protective effects, establishing a predictive framework for selecting potent honey types for hepatoprotection [107]. These findings underscore the need for further preclinical and clinical studies to elucidate the bioavailability, cellular signaling mechanisms, and region-specific bio-efficacy of Sri Lankan honeys, which could ultimately inform their strategic use in the management of oxidative stress-related liver disorders [102,103].

Table 6 provides a concise summary of preclinical and in vitro studies investigating the hepatoprotective effects of bee honey across different experimental models.

**Table 6:** Preclinical and in vitro studies on hepatoprotective effects of bee honey ADA: adenosine deaminase; AFP: alpha-fetoprotein; AFU: alpha-L-fucosidase; AGE: advanced glycation end products; ALT: alanine aminotransferase; ALP: alkaline phosphatase; AST: aspartate aminotransferase; BRL-3A: buffalo rat liver-3A (hepatocyte cell line); CCl₄: carbon tetrachloride; CAT: catalase; CNS: central nervous system; CPF: chlorpyrifos; DEN: diethylnitrosamine; DPPH: 2,2-diphenyl-1-picrylhydrazyl; FBS: fasting blood sugar; FST: forced swimming test; GGT: gamma-glutamyl transferase; GPx: glutathione peroxidase; GR: glutathione reductase; GSH: glutathione; GSH-Px: glutathione peroxidase; GST: glutathione S-transferase; HCC: hepatocellular carcinoma; HFD: high-fat diet; HIF-1α: hypoxia-inducible factor 1-alpha; HDL-C: high-density lipoprotein cholesterol; IL-1β: interleukin-1 beta; LDH: lactate dehydrogenase; LDL-C: low-density lipoprotein cholesterol; MAFLD: metabolic dysfunction-associated steatotic liver disease; MCP-1: monocyte chemoattractant protein-1; MDA: malondialdehyde; NASH: non-alcoholic steatohepatitis; NF-κβ: nuclear factor kappa beta; NLRP3: NLR family pyrin domain containing 3; NO: nitric oxide; NOS: nitric oxide synthase; Nrf2: nuclear factor erythroid 2–related factor 2; SD: Sprague-Dawley; SOD: superoxide dismutase; T1: treatment group 1; TAC: total antioxidant capacity; TBARS: thiobarbituric acid reactive substances; TC: total cholesterol; TGF-β1: transforming growth factor beta 1; TG: triglycerides; TNF-α: tumor necrosis factor-alpha; TPC: total phenolic content; TXNIP: thioredoxin-interacting protein

Study Type	Experimental Model	Type of Intervention	Key Outcomes	Mechanistic Insights	Reference
Experimental animal study	Male Wistar rats with carbon tetrachloride (CCl₄) -induced liver damage	20% honey samples (weak/medium/strong TPC/TAC) daily	↓ ALT, AST, ALP, FBS; ↓ serum and urinary MDA; ↑ thiol, DPPH; ↑ liver SOD, CAT, GPx; histology improved	Strengthens antioxidant defenses; modulates NO; suppresses inflammatory/fibrotic signaling; stabilizes hepatocyte membranes; phenolic content predicts potency	[107]
Experimental animal study	White rats subjected to forced swimming	Oral supplementation with Apis dorsata honey (2, 4, 6 g/rat/day for 14 days)	T1 (2 g/day) reduced liver damage; higher doses worsened injury	Moderate honey intake protects via antioxidant effects; excessive intake causes metabolic overload and oxidative imbalance	[108]
Experimental animal study	Adult female rats subjected to forced swimming test (FST)	Oral supplementation with Indian forest bee honey (2, 4, 6 g/day for 14 days)	↓ hepatic HIF-1α and TNF-α expression, ↑ hepatic SOD expression (not statistically significant)	Honey modulates oxidative stress markers; reduces hypoxia-induced and inflammatory stress; enhances antioxidant defense	[109]
Experimental animal study	Male Wistar rats, alloxan-induced diabetes	Natural honey (0.2, 0.5, 0.8 mL/day for 21 days)	↓ blood glucose; improved lipid profile (↓ TC, TG, LDL-C; ↑ HDL-C); normalized ALT, AST, ALP, bilirubin	Antioxidant-mediated hepatoprotection; lipid metabolism modulation; stabilizes liver enzymes; dose-dependent effects comparable to Glibenclamide	[110]
Experimental animal study	Male Sprague–Dawley rats with HFD-induced MAFLD	Oral bee bread (0.5 g/kg/day for 12 weeks)	↓ serum lipids and liver enzymes; ↑ antioxidant enzymes; ↓ TBARS, NO; ↓ TNF-α, NF-κβ, MCP-1; improved histopathology	Activates Nrf2 signaling, enhances antioxidant defense; downregulates NF-κβ; reduces hepatic lipid accumulation, inflammation, and fibrosis	[106]
Experimental animal study	Mice with bromobenzene-induced liver damage	Oral Apis cerana honey (dose-dependent, high dose most effective)	↓ ALT (59.13%), ↓ AST (79.71%); ↓ hepatic MDA (63.30%); ↑ SOD (73.12%), ↑ GSH-Px (57.24%); ↓ TGF-β1 (51.83%)	Polyphenols and antioxidants reduce oxidative stress; upregulate SOD/GSH-Px; downregulate TGF-β1 → antifibrotic, anti-inflammatory hepatoprotection	[105]
Experimental animal study	Mice with acute alcohol-induced liver damage	Oral pretreatment with Apis cerana honey (5, 10, 20 g/kg, twice daily for 12 weeks)	↓ ALT, AST; ↓ hepatic MDA; ↑ SOD, GSH-Px; ↓ TGF-β1	Antioxidant compounds (phenolics, vitamin C) scavenge free radicals; inhibit lipid peroxidation; upregulate antioxidant enzymes; downregulate fibrogenic signaling	[104]
Experimental animal study	Rats, HCC induced by DEN + carbon tetrachloride (CCl₄)	Natural bee honey (post-induction; dose not specified)	↓ AST, ALT, ALP, GGT; ↓ lipid peroxidation; ↑ SOD, CAT, GPx, GST, GSH; improved histoarchitecture; ↓ AFP and AFU	Antioxidant, anti-inflammatory, anticancer activity; restores enzymatic/non-enzymatic defenses; prevents oxidative stress-driven liver injury and tumorigenesis	[111]
Experimental animal and cell culture study	Female Sprague–Dawley rats (NASH, HFD) + BRL-3A hepatocytes (sodium palmitate-induced steatosis)	Honey (5 g/kg/day in rats; in vitro pre-treatment)	In vivo: ↓ hepatic injury, steatosis, fibrosis, oxidative stress and inflammation; inhibited TXNIP and NLRP3. In vitro: suppressed NLRP3 activation, restored lipid metabolism	Downregulates TXNIP; suppresses NLRP3 inflammasome; reduces oxidative stress and inflammatory signaling; restores lipid homeostasis	[112]
Experimental animal study	Rats, aflatoxin-induced toxicity	Natural honey (dose unspecified)	↓ AST, ALT, GGT; ↓ lipid peroxidation; ↑ GSH, GR, SOD, GST, CAT; histology protected	Enhances enzymatic antioxidant defenses; stabilizes hepatocyte membranes; multiorgan protection	[113]
Experimental animal study	Female Wistar rats, CPF-induced toxicity	Natural honey (3 g/kg/day, 4 weeks)	Restored liver and kidney biochemical parameters; ↓ lipid peroxidation; improved body and liver weight; histology improved	Antioxidant effect; restores protein metabolism; prevents hepatic necrosis and inflammation; protection against CPF-induced oxidative stress	[114]
Experimental animal study	Male albino rats, melamine-induced hepatotoxicity	Natural bees’ honey (2.5 g/kg, 28 days post-melamine)	↓ ALT, AST, ALP; ↑ total protein; restored hepatic morphology	Antioxidant and membrane-stabilizing effects; ameliorates protein metabolism disturbances; histopathological protection	[115]
Experimental animal study	Rats, metanil yellow-induced hepatotoxicity	Bees’ honey (2.5 mg/kg/day, 8 weeks)	↓ oxidative stress; ↓ AGE and NF-κB; ↓ TNF-α, IL-1β; histology protected	Antioxidant action; anti-inflammatory via NF-κB modulation; hepatocyte structural protection	[116]
Experimental animal study	Male albino rats, lead acetate toxicity	Natural honey (1.5 mL/kg/day, 4 weeks)	↓ MDA; ↑ GPx and NO; ↓ AST, ALT, ALP; ↓ urea, creatinine; improved histology	Antioxidant activity; protects liver and kidney from oxidative injury; stabilizes tissue integrity	[117]
Experimental animal study	Male rats with obstructive jaundice	Oral honey supplementation	↓ ALT and ADA; ↑ NO in erythrocytes and liver	Hepatocellular protection via NO-mediated pathways; antioxidant and hepatoprotective effects	[11]

Discussion

The global burden of liver diseases continues to rise, with conditions such as acute hepatitis, alcoholic liver disease, cirrhosis, and NAFLD contributing significantly to morbidity and mortality. These disorders are often underpinned by a triad of pathological processes: oxidative stress, chronic inflammation, and hepatocyte apoptosis. Conventional pharmacotherapies, while effective in certain contexts, are frequently limited by adverse effects, cost, and accessibility, particularly in low-resource settings. This has prompted growing interest in plant-based and integrative approaches, including polyherbal formulations rooted in traditional medicine systems. Within this landscape, Sri Lankan-origin standardized polyherbal formulations, based on Ayurvedic principles, emerge as promising candidates for hepatoprotection, warranting systematic exploration. This scoping review systematically mapped the hepatoprotective potential of the individual ingredients in such formulations, *O. octandra*, *T. indica, P. nigrum, P. longum, C. verum*, and bee honey, revealing a robust foundation for their therapeutic development.

Potential Mechanisms of Action of the Sri Lankan-Origin Polyherbal Formulation in Liver Protection

**Figure 4:**
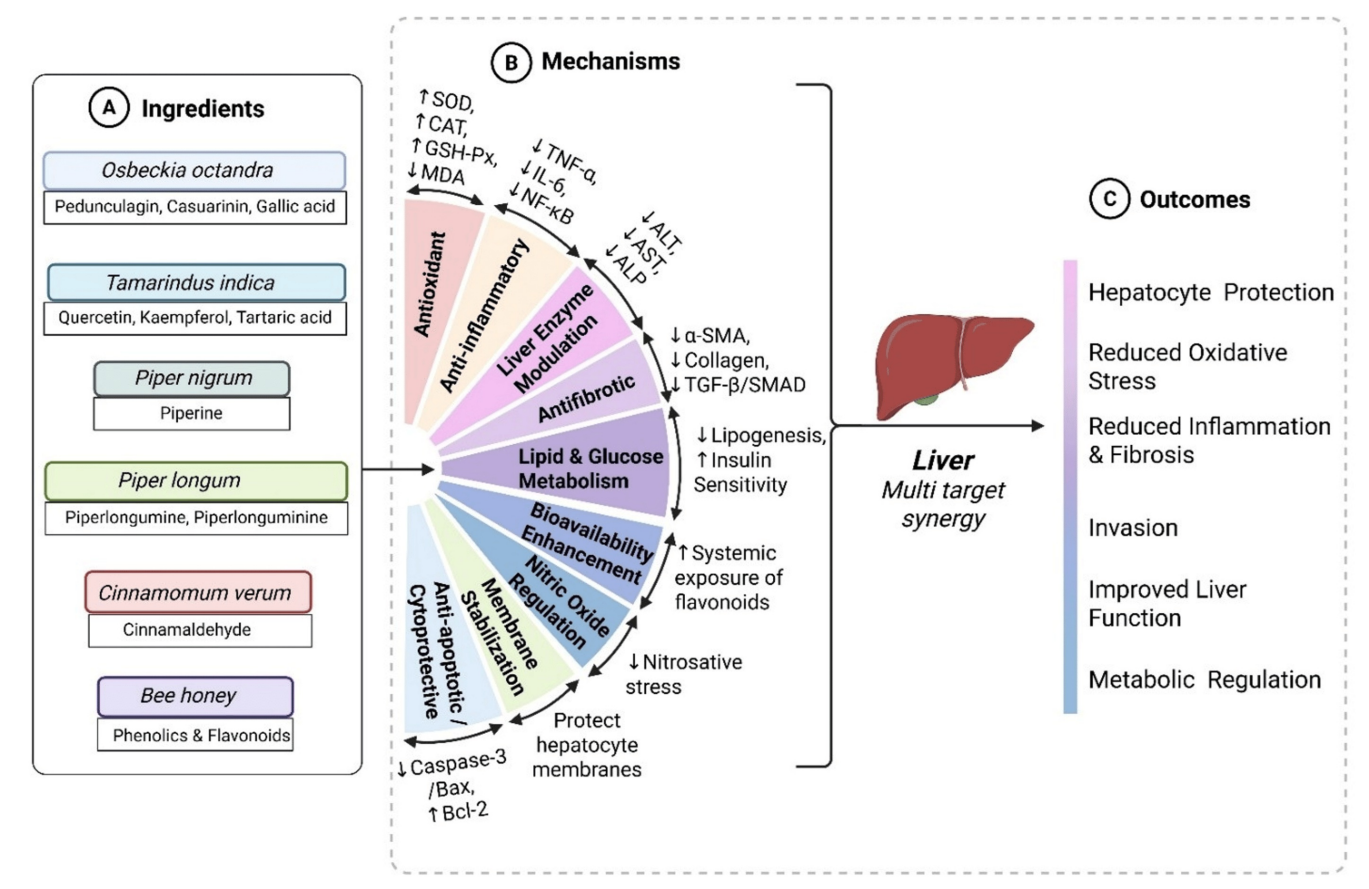
Overview of potential mechanisms of action in liver protection by a Sri Lankan-origin standardized polyherbal formulation Image Credit: Kalmee P. Kariyawasam (author) SOD: superoxide dismutase; CAT: catalase; GSH-Px: glutathione peroxidase; MDA: malondialdehyde; TNF-α: tumor necrosis factor alpha; IL-6: interleukin-6; NF-κB: nuclear factor kappa-light-chain-enhancer of activated B cells; ALP: alkaline phosphatase; ALT: alanine aminotransferase; AST: aspartate aminotransferase; α-SMA: alpha-smooth muscle actin; TGF-β1: transforming growth factor beta 1

The Sri Lankan-origin standardized polyherbal formulation appears to exert hepatoprotective effects through a constellation of biochemical and molecular pathways. Its ingredients collectively contribute to a multi-target therapeutic profile. As illustrated in Figure 4, the formulation’s bioactive compounds engage antioxidant, anti-inflammatory, anti-apoptotic, and enzyme-modulating activities, while also enhancing the bioavailability of co-administered phytochemicals.

Antioxidant Activity

Oxidative stress is a primary driver of liver injury, and this polyherbal formulation contains multiple bioactive compounds that counteract this process by enhancing endogenous antioxidant defenses. Pedunculagin and casuarinin from* O. octandra* significantly upregulate superoxide dismutase, catalase, and glutathione peroxidase, while reducing lipid peroxidation markers such as malondialdehyde, thereby preserving hepatocyte integrity [13]. Similarly, *T. indica* contributes quercetin and kaempferol, which scavenge reactive oxygen species and restore redox balance in models of drug-induced liver injury [39]. Piperine from *P. nigrum* and piperlongumine from *P. longum* also enhance antioxidant enzyme activity and reduce oxidative damage in hepatocytes (56,72). Bee honey derived from *A. cerana* and *A. dorsata* adds further antioxidant support through its rich phenolic and flavonoid content, boosting enzymatic antioxidants and reducing oxidative stress markers [104].

Anti-inflammatory Effects

Chronic hepatic inflammation contributes to fibrosis and disease progression, and several constituents of the formulation exhibit potent anti-inflammatory properties. Gallic acid from *O. octandra *suppresses pro-inflammatory cytokines such as TNF-α and IL-6 and downregulates fibrogenic markers, including vascular endothelial growth factor receptor 2 and alpha-smooth muscle actin [13]. Quercetin from* T. indica* reduces tumor necrosis factor-alpha levels and improves histopathological outcomes in thioacetamide-induced liver injury [40]. Piperine from *P. nigrum* inhibits nuclear factor kappa-light-chain-enhancer of activated B cells (NF-κB) signaling and reduces inflammatory cytokine expression, contributing to both anti-inflammatory and antifibrotic effects [57]. Piperlonguminine from *P. longum* further suppresses collagen deposition and inflammatory mediators in fibrotic models [69]. Cinnamaldehyde from *C. verum *modulates inflammatory gene expression and reduces serum TNF-α and IL-6 levels [81], while bee honey inhibits transforming growth factor beta 1 and TNF-α, reducing inflammation in cholestatic liver injury [107].

Liver Enzyme Modulation

Normalization of liver enzymes is a key indicator of hepatoprotection, and several ingredients of this formulation have demonstrated efficacy in modulating these biomarkers. Quercetin and tartaric acid from *T. indica* significantly reduce serum levels of ALT, AST, and ALP, indicating improved liver function [41]. Piperine from *P. nigrum* and piperlongumine from *P. longum* restore liver enzyme profiles in models of acetaminophen- and antiretroviral-induced hepatotoxicity [62,72]. Cinnamaldehyde from *C. verum* consistently reduces ALT and AST levels in both preclinical and clinical studies, including trials involving NAFLD patients [90]. Bee honey also contributes to enzyme normalization, with studies reporting reductions in ALT and improvements in histological liver grades [106].

Antifibrotic Activity

Antifibrotic activity is particularly evident in compounds like pedunculagin and gallic acid from *Osbeckia octandra*, which inhibit fibrogenic signaling pathways such as transforming growth factor beta/SMAD, leading to downregulation of Alpha-smooth muscle actin and Vascular endothelial growth factor receptor 2 expression [13]. Piperlongumine from Piper longum also demonstrates antifibrotic effects by reducing collagen deposition and suppressing hepatic stellate cell activation [69]. These actions are critical in preventing progression from chronic inflammation to cirrhosis.

Modulation of Lipid and Glucose Metabolism

Modulation of lipid and glucose metabolism is another mechanism relevant to metabolic liver diseases like MAFLD. Piperlongumine and its derivatives regulate transcription factors such as cAMP response element-binding protein and sterol regulatory element-binding protein 1c, thereby reducing hepatic gluconeogenesis and lipogenesis [70]. Similarly, *T. indica* extracts have shown improvements in lipid profiles and insulin sensitivity, suggesting a role in metabolic regulation alongside hepatoprotection [41].

Bioavailability Enhancement

One of the unique pharmacological advantages of the Sri Lankan-origin standardized polyherbal formulation is its potential to enhance the bioavailability of co-administered phytochemicals. Piperine, an alkaloid present in both *P. nigrum* and *P. longum*, inhibits hepatic and intestinal glucuronidation, thereby increasing systemic exposure to other bioactive compounds such as flavonoids and polyphenols [64]. This effect is particularly relevant for compounds like quercetin and kaempferol from *T. indica*, which otherwise suffer from poor oral bioavailability. By modulating drug-metabolizing enzymes and transporters, piperine enhances the therapeutic efficacy of the formulation without requiring higher doses, supporting its inclusion as a bioenhancer within the polyherbal supplement.

Nitric Oxide Regulation

Nitric oxide regulation is highlighted in studies involving bee honey, particularly from *A. cerana *and *A. dorsata*. Honey modulates nitric oxide synthase activity and reduces nitrosative stress, which is implicated in cholestatic and drug-induced liver injury [11]. This mechanism complements its antioxidant and anti-inflammatory effects, contributing to vascular and cellular stability within hepatic tissue.

Membrane Stabilization

Membrane stabilization is a recurring theme across several ingredients. Ellagitannins from *O. octandra* and flavonoids from bee honey preserve hepatocyte membrane integrity, preventing leakage of liver enzymes and maintaining cellular architecture [25,106]. Piperine also contributes to membrane stabilization, particularly in models of antiretroviral-induced hepatotoxicity [62].

Anti-apoptotic and Cytoprotective Activity

Preventing hepatocyte apoptosis is essential for liver regeneration and recovery. Kaempferol from *T. indica* and cinnamaldehyde from *C, verum* modulate apoptotic gene expression by reducing pro-apoptotic markers such as caspase-3 and Bcl-2-associated X protein, while upregulating anti-apoptotic proteins like B-cell lymphoma 2 (Bcl-2) [48,96]. Piperine from *P. nigrum* stabilizes hepatocyte membranes and reduces apoptosis in models of drug-induced liver injury [62]. Piperlongumine from *P. longum *influences cAMP response element-binding protein and sterol regulatory element-binding protein 1c signaling, reducing lipogenesis and apoptosis [72]. Bee honey also exhibits cytoprotective effects, with its flavonoids and enzymatic antioxidants preserving membrane integrity and reducing hepatocyte death in MAFLD models [106].

Potential Synergistic Effects of Combining Ingredients

While the individual ingredients of the Sri Lankan-origin standardized polyherbal formulation have each demonstrated hepatoprotective efficacy through distinct mechanisms, the potential for synergistic interactions within the complete formulation remains a scientifically compelling but empirically unvalidated area. Polyherbal synergy may arise through complementary pharmacodynamics, enhancement of bioavailability, or mitigation of toxicity; however, such effects must be rigorously demonstrated in experimental and clinical studies.

One of the most well-supported mechanisms of synergy is bioavailability enhancement mediated by piperine, the principal alkaloid in *P. nigrum* and *P. longum*. Piperine inhibits hepatic and intestinal glucuronidation, thereby increasing systemic exposure to co-administered phytochemicals such as flavonoids and polyphenols [62]. This effect has been shown to potentiate the hepatoprotective actions of curcumin, quercetin, and other poorly absorbed compounds. Within the LivosBEE™ context, such a mechanism could enhance the antioxidant and anti-inflammatory effects of polyphenols from *T. indica *and *C. verum*, which are otherwise limited by poor oral bioavailability [48,96].

Beyond pharmacokinetics, pharmacodynamic synergy may occur through overlapping antioxidant and anti-inflammatory activities. For example, *O. octandra *and bee honey are rich in flavonoids and phenolic compounds, which independently upregulate antioxidant enzymes (superoxide dismutase, catalase, glutathione peroxidase) while downregulating oxidative stress and inflammatory markers such as malondialdehyde and TNF-α [13,104,107]. Their combined use may therefore lead to additive or synergistic free radical scavenging and cytokine suppression, particularly in chemically induced models of hepatic injury. Similarly, cinnamaldehyde from *C. verum* and piperlongumine from *P. longum *both modulate apoptotic gene expression, downregulating Bcl-2-associated X protein and Caspase-3, while upregulating Bcl-2, suggesting possible synergy in protecting hepatocytes against apoptosis [72,96].

Nonetheless, synergy is not guaranteed and may be influenced by dose ratios, extraction methods, phytochemical stability, and pharmacokinetic interactions. For instance, excessive honey intake has been reported to reverse hepatoprotective effects due to metabolic overload, underscoring the need for dose optimization [108]. Likewise, while piperine improves bioavailability, it can also alter hepatic enzyme activity and drug metabolism, raising the possibility of adverse interactions [63,64].

At present, none of the reviewed studies have directly evaluated the complete LivosBEE™ formulation in either in vitro or in vivo settings. To substantiate the hypothesis of synergy, future research should employ isobolographic analysis, combination index models, and systems pharmacology approaches to quantify interaction effects. Transcriptomic and metabolomic profiling could further clarify whether multi-ingredient formulations activate convergent or divergent pathways in hepatoprotection. The pharmacological rationale for synergy in LivosBEE™ is persuasive, particularly with respect to bioavailability enhancement by piperine and overlapping antioxidant mechanisms from polyphenol-rich ingredients, but empirical validation through controlled formulation-level studies is essential before therapeutic claims can be substantiated.

Safety Profile of the Ingredients of the Sri Lankan-Origin Polyherbal Formulation

The six core ingredients of the Sri Lankan-origin standardized polyherbal formulation have long-standing ethnomedical relevance and are generally regarded as safe when consumed within recommended limits. Toxicological assessments indicate that *T. indica* and *C. verum *possess high LD₅₀ values (>5000 mg/kg), categorizing them as practically non-toxic in acute exposure models [118,119]. Tamarind is also regarded as GRAS (Generally Recognized as Safe) with no evidence of acute toxicity. Ceylon cinnamon (*C. verum*), unlike its *cassia* counterpart, contains markedly lower levels of coumarin (<100 mg/kg bark), minimizing the risk of hepatotoxicity when consumed in moderation [120].

*O. octandra *has not been assigned an LD₅₀ value, yet available in vitro studies demonstrate low cytotoxicity and no reported adverse effects, supporting its classification as generally safe. Bee honey, widely consumed and valued for its antioxidant properties, requires quality assurance to control 5-hydroxymethylfurfural (HMF), a contaminant formed during heating. Elevated HMF levels (>80 mg/kg) may indicate poor-quality honey, particularly in tropical regions. Caution is warranted in special populations: honey should not be given to infants under 12 months due to the risk of botulism, and diabetic individuals should consume it in moderation.

The safety of *P. nigrum* and* P. longum*, both rich in piperine, is dose-dependent. Their LD₅₀ values in mice range between 330 and 514 mg/kg, while the recommended safe daily dose for adults is approximately 14 mg (0.2 mg/kg for a 70-kg individual). Higher doses (≥50 mg/kg) have been associated with adverse effects, and even low levels (≈5 mg/day) may interact with concomitant medications by altering hepatic drug metabolism [121]. Contraindications include pregnancy, breastfeeding, bleeding disorders, and diabetes, emphasizing the need for caution in vulnerable populations.

Collectively, the ingredients of the formulation demonstrate favorable safety profiles when used appropriately and within recommended limits. Nevertheless, formulation-level safety must be established through standardized dosing regimens, stringent quality control, and clinical evaluation. Future investigations should assess cumulative toxicity, pharmacokinetic interactions, and long-term safety to ensure the suitability of the polyherbal supplement for broader therapeutic application.

The safety of the Sri Lankan-origin standardized polyherbal formulation is thus supported by both traditional usage and toxicological evidence (Table 7).

**Table 7:** Toxicity levels of the ingredients of the Sri Lankan-origin polyherbal formulation GRAS: generally recognized as safe; HMF: 5-hydroxymethylfurfural

Herb/Substance Name	LD50 Value	Safe Dose Limit	Toxic Threshold	Contraindications	Reference
Osbeckia octandra (Heen Bowitiya)	Not established	Low cytotoxicity demonstrated in vitro	No reported toxicity	Generally safe	[7]
Piper nigrum (Black Pepper/Gammiris) - Piperine	330-514mg/kg (oral, mice)	Maximum 14 mg/day for adults (0.2 mg/kg for 70kg adult)	>50 mg/kg shows adverse effects; 5 mg/day may cause drug interactions	Pregnancy, breastfeeding, blood clotting disorders, diabetes, concurrent medications	[55,121]
Piper longum (Long Pepper/Thippili) - Piperine	330-514mg/kg (oral, mice)	Maximum 14 mg/day for adults (0.2 mg/kg for 70kg adult)	>50 mg/kg shows adverse effects; 5 mg/day may cause drug interactions	Pregnancy, breastfeeding, blood clotting disorders, diabetes, concurrent medications	[55,121]
Tamarindus indica (Tamarind /Siyambala)	>5000 mg/kg (Practically non-toxic)	Generally recognized as safe (GRAS)	No reported acute toxicity	Generally safe	[119]
Cinnamomum verum (Ceylon Cinnamon/Kurundu)	>5000 mg/kg (practically non-toxic)	0.1 mg coumarin/kg body weight/day	Ceylon: <100 mg coumarin/kg bark (vs Cassia: 2650-7017 mg/kg)	Long-term high doses of coumarin may cause liver toxicity	[118, 120]
Bee Honey –5-hydroxymethylfurfural (HMF)	Not established	<40 mg/kg (depending on region – 80 mg/kg for honey that originates from countries or regions with tropical temperatures)	Varies by contaminant (e.g., HMF >80 mg/kg indicates poor quality)	Never give to infants <12 months (botulism risk); monitor in diabetes	[102,117]

Critical Appraisal and Future Directions

The body of evidence reviewed offers valuable insights into the hepatoprotective properties of the individual ingredients of Sri Lankan-origin standardized polyherbal formulations, yet it also reveals significant limitations that must be addressed to advance such formulations toward clinical application. A key strength lies in the diversity of experimental models employed across studies, encompassing chemically induced hepatotoxicity (e.g., CCl_4_, thioacetamide, paracetamol), metabolic liver disease (e.g., NAFLD), and drug-induced injury (e.g., anti-tubercular and antiretroviral agents). This breadth enhances the generalizability of findings across liver pathologies and reflects the multifactorial nature of hepatic disorders. Moreover, the ethnobotanical relevance of the ingredients, particularly *O. octandra* and bee honey from Sri Lankan sources, adds cultural and historical depth, aligning the formulation with traditional South Asian medicinal practices.

Although the current evidence provides valuable insights into the hepatoprotective potential of these individual ingredients, a few limitations should be acknowledged. Some heterogeneity exists across studies, particularly with respect to animal models, hepatotoxic induction methods, extraction types (aqueous, ethanolic, methanolic), and dosing regimens, which makes direct comparison more complex. In addition, while many investigations are promising, only a limited number included phytochemical profiling or batch standardization, which would strengthen reproducibility. Clinical data are still relatively scarce, and none to date have assessed the complete polyherbal formulation, although preclinical models provide a useful foundation. Most experimental studies used small sample sizes or short intervention periods, and longer-term effects remain to be explored. Finally, randomized controlled trials and detailed mechanistic investigations at the formulation level are still lacking, offering important opportunities for future research.

To bridge these gaps, future research must prioritize clinical validation through well-designed trials. Phase I studies should assess safety, tolerability, and dose optimization, followed by Phase II/III trials targeting specific liver conditions such as NAFLD, drug-induced hepatotoxicity, or viral hepatitis. Standardization of extraction protocols and rigorous phytochemical profiling are essential to ensure batch-to-batch consistency, guided by frameworks from the WHO and AYUSH (Ayurveda, Yoga & Naturopathy, Unani, Siddha, and Homoeopathy). Mechanistic elucidation should leverage advanced techniques such as transcriptomics, proteomics, and metabolomics, enabling systems biology modeling of multi-target interactions. Formulation optimization, including exploration of delivery systems like nano-formulations or liposomal encapsulation, may enhance bioavailability and therapeutic efficacy. The role of piperine as a bioenhancer should be systematically investigated within these designs. Finally, integrating ethnopharmacological knowledge with modern pharmacology can foster culturally sensitive and scientifically robust interventions. Community engagement and documentation of traditional usage patterns may inform formulation refinement and support regulatory acceptance.

## Conclusions

This scoping review consolidates the evidence for a Sri Lankan-origin standardized polyherbal formulation composed of *O. octandra,* *T. indica, P. nigrum, P. longum, C. verum*, and bee honey. The antioxidant, anti-inflammatory, and anti-apoptotic constituents showed strong activity, modulation of aminotransferase, and suppression of lipid peroxidation and inflammatory signaling, upregulated endogenous antioxidant enzymes, and the histological preservation of hepatic architecture in different preclinical models, even though limited evidence exists for beneficial clinical use. The pharmacological variations in this compound are the source for a plausible multicomponent justification that not only has polyphenolic and ellagitannin components, including *O. octandra and T. indica, *for redox and cytokine modification but also for cinnamaldehyde/eugenol rich fractions of *C. verum* for membrane stability and anti-apoptotic activity, bee honey contributing phenolics with antifibrotic and redox modulation, and the piperamides especially piperine, taken from *P. nigrum, and P. longum,* which can exert direct anti-inflammatory actions as well as an effective bioenhancement mechanism through glucuronidation inhibition that could possibly raise systemic level of co-administered phytochemicals. Collectively, these features provide a framework for formulation-level synergy.

However, critical gaps remain. No study evaluated the full formulation in vitro or in vivo; thus, the claim of synergy and clinical efficacy must be tentative. Priorities will be (i) standardized extraction, phytochemical profile, and batch-to-batch characterization; (ii) dose-finding/safety and tolerability (Phase I) measures and (iii) disease-centric efficacy studies (Phase II/III) with mechanistic endpoints (transcriptomics/metabolomics) and quantitative interaction methods (such as isobolography or combination-index modeling). Indications for safety at the level of ingredients seem to be acceptable within standard ranges, although there are some limitations concerning piperine-drug interactions, honey quality control (HMF), and potential population restrictions; formulation-related pharmacokinetic interactions and cumulative-toxicity evaluations need to be made. The final clinical utility will largely be determined by in-depth validation of the final formulation, titration to optimal dosage, and harmonization with regulations to guarantee reproducibility, safety, and efficacy.
